# Crotonate suppresses breast cancer metastasis and promotes immunotherapy response by inducing ACSS2-mediated EZH2-K348 crotonylation

**DOI:** 10.1126/sciadv.aea9892

**Published:** 2026-01-16

**Authors:** Bo Liu, Xinwei Duan, Ge Wang, Youzhi Tang, Kunhao Zhou, Jing Zhang, Yu Yu, Hongquan Zhang

**Affiliations:** ^1^ Department of Human Anatomy, Histology and Embryology, School of Basic Medical Sciences, PKU International Cancer Institute, Peking University Health Science Center, Beijing 100191, China.; ^2^ Department of Obstetrics and Gynecology, Peking University First Hospital, Beijing 100034, China.

## Abstract

Crotonate, a short-chain fatty acid, generates protein crotonylation. However, the role of crotonate in cancer progression is unknown. Here, we present a crotonate–crotonyl–coenzyme A (CoA)–enhancer of zeste homolog 2 (EZH2) crotonylation cascade blocking breast cancer growth and metastasis. We demonstrated that crotonate promotes EZH2 degradation via crotonyl-CoA–mediated crotonylation at Lys^348^ in EZH2 (EZH2-K348cr). EZH2-K348cr leads to reduced genome-wide H3K27me3 (trimethylation of lysine-27 on histone-3) occupancy. Crotonate metabolizes to crotonyl-CoA by ACSS2 (acyl-CoA synthetase 2), and then, acyltransferase p300 catalyzes crotonyl-CoA and generates EZH2-K348cr. Crotonylated EZH2 triggers EZH2 ubiquitination and degradation. Administration of crotonate markedly inhibits breast cancer cell growth and metastasis via a crotonate-crotonyl-CoA-EZH2-K348cr cascade. In comparison, crotonate showed better blocking effect than EZH2 inhibitor tazemetostat in suppressing breast cancer metastasis. The combination of crotonate and anti-PD-L1 (programmed cell death ligand 1) antibody enhances responses of breast cancer cells to immunotherapy. Together, our findings indicate that crotonate is a promising anticancer drug candidate that suppresses breast cancer growth and metastasis by specifically inducing EZH2 degradation.

## Teaser

Short-chain fatty acid crotonate is a possible breast cancer killer through inducing the degradation of oncogenic protein EZH2.

## INTRODUCTION

Fatty acids are classified into three categories based on chain length: long-chain fatty acids (LCFAs), medium-chain fatty acids (MCFAs), and short-chain fatty acids (SCFAs). SCFAs, which are monocarboxylic acids containing fewer than six carbon atoms, primarily consist of acetate, propionate, butyrate, crotonate, and valerate. As key microbial metabolites, SCFAs regulate gene expression by inhibiting histone deacetylase (HDAC) activity and promoting histone acylation modification (*1*–*3*). Specifically, both propionate and butyrate enhance chromatin accessibility through the acylation of specific genomic regions, thereby driving gene expression (*4*). Butyrate, a potent HDAC inhibitor, has been shown to suppress the proliferation of cancerous colonocytes (*5*). In addition, butyrate inhibits liver metastasis in colorectal cancer cells and lung metastasis in breast cancer cells (*6*, *7*). High-fiber diets, which enhance SCFA production, may provide clinical benefits for treating estrogen receptor–positive, endocrine-resistant breast cancer patients (*8*). Recently, an opposing role of SCFAs has been reported in non–small cell lung cancer. Dietary acetate supplementation not only promotes tumor growth but also diminishes CD8^+^ T cell infiltration (*9*). However, the role of crotonate, a less well-studied SCFA, in cancer progression and whether dietary crotonate supplementation could serve as a promising preventive or therapeutic strategy remain largely unexplored.

During fatty acid metabolism, fatty acids are activated by acyl–coenzyme A (CoA) synthetases to form acyl-CoA, which enables β-oxidation. Crotonate is the direct precursor of crotonyl-CoA, which can also be derived from butyryl-CoA or glutaryl-CoA and subsequently oxidized to acetyl-CoA for entry into the tricarboxylic acid cycle. Crotonyl-CoA serves as a donor for histone lysine crotonylation (Kcr) to facilitate gene transcription (*10*, *11*). Kcr is dynamically regulated by histone acetyltransferases (HATs) and HDACs (*12*, *13*). Enzymes such as crotonyl-CoA synthetase ACSS2 (acyl-CoA synthetase 2), crotonyl-CoA hydratase CDYL (chromodomain Y–like protein), and other acyl-CoA synthetases also function in modulating Kcr. In addition to histones, numerous nonhistone proteins undergo Kcr modification, thereby regulating protein activity, localization, and stability (*14*–*16*). Although Kcr is involved in various physiological and pathological processes, the role of crotonyl-CoA in cancer metastasis and the underlying mechanisms remain poorly understood.

The histone methyltransferase (HMT) enhancer of zeste homolog 2 (EZH2) serves as the enzymatic catalytic subunit of the polycomb repressive complex 2 (PRC2). PRC2 suppresses gene expression by catalyzing the trimethylation of lysine-27 on histone-3 (H3K27me3), predominantly targeting tumor suppressor genes. Multiple postmodification of EZH2 affects its activity and stability such as phosphorylation, glycosylation, acetylation, and methylation (*17*). EZH2 plays a critical role in promoting tumor growth, metastasis, metabolism, and regulating antitumor immunity. Both overexpression and mutations of EZH2 have been implicated in the progression of various cancers, highlighting its potential as a promising therapeutic target (*18*, *19*). EZH2 is frequently overexpressed in breast cancer, and high expression levels are strongly associated with poor prognosis (*20*). EZH2 inhibition has been shown to up-regulate programmed cell death ligand 1 (PD-L1) expression and increase the infiltration of CD8^+^ T cells in prostate cancer and colon cancer (*21*, *22*). Tumors with enhanced EZH2 activity or stability frequently display immunosuppressive tumor microenvironments and are often resistant to immunotherapy (*23*, *24*).

In this study, we aim to unravel the role of crotonate metabolism in breast cancer progression. We found that crotonyl-CoA derived from crotonate mediates the crotonylation of EZH2 at Lys^348^, leading to destabilization and subsequent degradation of EZH2. Supplementation of crotonate effectively inhibits breast cancer progression. The combination of crotonate and immune checkpoint blockade therapy exhibits a synergistic anticancer effect. Therefore, targeting EZH2 degradation through crotonate or crotonyl-CoA supplementation represents a promising therapeutic strategy for breast cancer treatment.

## RESULTS

### Crotonate suppresses breast cancer cell proliferation and migration

**Fig. 1.**
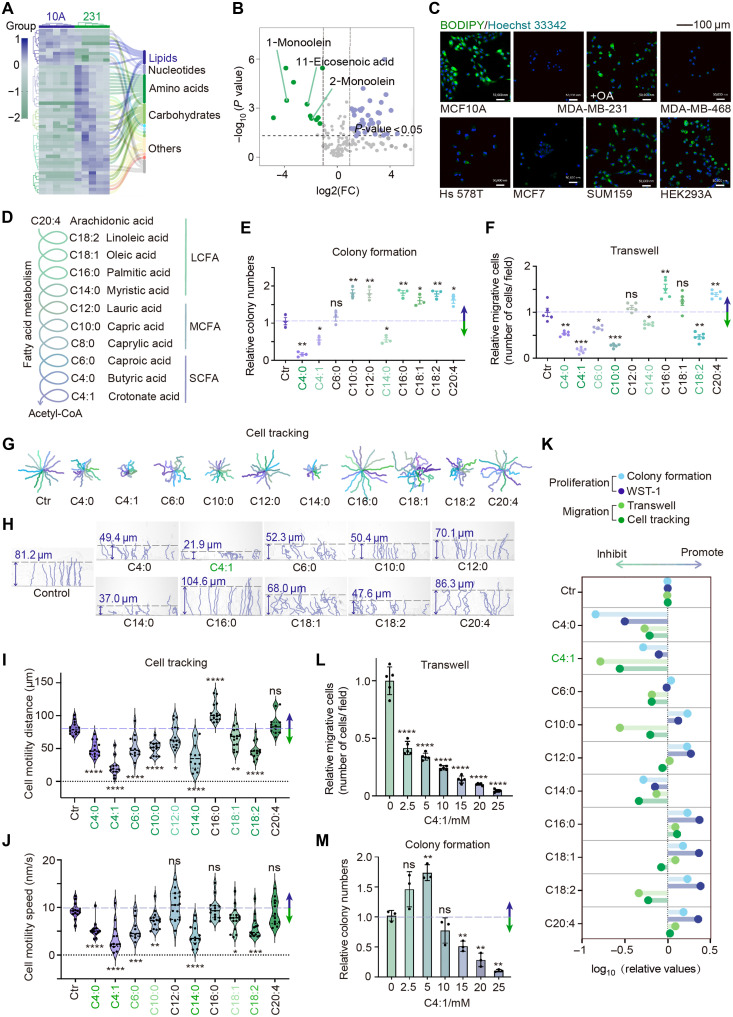
Fatty acids regulate cancer cell proliferation and motility. (**A**) Gas chromatography–mass spectrometry analysis in MCF10A and MDA-MB-231 cells (*n* = 5). The top 50 differential metabolites [*P* < 0.05, fold change (FC) > 2.0] were selected for hierarchical clustering analysis. (**B**) Volcano plot shows the differential metabolites. (**C**) Immunofluorescence staining in different cell lines and oleic acid (0.1 mM, 72 hours)–treated MDA-MB-231 cells using BODIPY (green) and Hoechst 33342 (blue). (**D**) Schematic diagram of fatty acid catabolic pathways. (**E** to **J**) MDA-MB-231 cells were treated with various fatty acids for 72 hours, and followed by colony formation (E), transwell (F), and TAXIScan-FL (G to J). The fatty acid we used are C4:0 (10 mM), C4:1 (20 mM), C6:0 (5 mM), C10:0 (0.1 mM), C12:0 (0.1 mM), C14:0 (0.1 mM), C16:0 (0.1 mM), C18:1 (0.1 mM), C18:2 (0.1 mM), and C20:4 (0.1 mM). The movement trajectories of individual cells (G and H) and the distance and speed of cell motility for each group (I and J) were analyzed. Data represent the means ± SEM (E) or ± SD (F). *P < 0.05; **P < 0.01; ***P < 0.001; ****P < 0.0001, unpaired *t* test with Welch’s correction. Ctr, control; ns, not significant. (**K**) Systemic analysis of the effect of different fatty acids on cell proliferation and migration based on the average colony numbers (E), the average cellular viability on the fifth day in WST-1 experiment (fig. S1, A and B), the average number of migrative cells in transwell assay (F), and the average motility distance (I). (**L** and **M**) MDA-MB-231 cells were treated with different concentrations of crotonate for 72 hours, followed by transwell (L) and colony formation (M) assays. Data represent the means ± SD (L) or ± SEM (M). **P < 0.01; ****P < 0.0001, unpaired *t* test with Welch’s correction.

To elucidate the metabolic characteristics of breast cancer cells, we conducted an untargeted metabolomic analysis on both the human breast cancer cell line MDA-MB-231 and the human normal breast epithelial cell line MCF10A. Our results revealed that breast cancer cells exhibited reduced levels of lipid metabolites and elevated levels of amino acids and carbohydrates compared to normal breast epithelial cells (Fig. 1A). Specifically, among the down-regulated lipid metabolites, 11-eicosenoic acid, 1-monoolein, and 2-monoolein, which are known fatty acid metabolism products, were notably decreased (Fig. 1B). In addition, boron-dipyrromethene (BODIPY) staining demonstrated a markedly greater abundance of lipid droplets in normal breast epithelial cells compared to breast cancer cells (Fig. 1C). These findings suggest that lipid metabolism, particularly fatty acid metabolism, is less active in breast cancer cells, indicating that fatty acid metabolism may exert a suppressive effect on breast cancer progression.

During fatty acid catabolism, fatty acids undergo β-oxidation, which sequentially converts LCFAs into progressively shorter chains, ultimately yielding MCFAs and SCFAs (Fig. 1D). To identify the most effective fatty acids in inhibiting cancer progression, we systematically evaluated the effects of different fatty acids on key cellular processes, including proliferation, migration, and motility. First, we assessed the impact of various fatty acids on breast cancer cell proliferation through water-soluble tetrazolium salt 1 (WST-1) and colony formation assays. The results indicated that the SCFAs butyric acid and crotonic acid, as well as the LCFA myristic acid, inhibited breast cancer cell proliferation, whereas most MCFAs and LCFAs promoted cell proliferation (Fig. 1E and fig. S1, A and B). Furthermore, transwell assays revealed that three SCFAs, the MCFA decanoic acid, and the LCFAs myristic acid and linoleic acid suppressed breast cancer cell migration, whereas the LCFAs palmitic acid and arachidonic acid exhibited a promoting effect (Fig. 1F). Moreover, we used the TAXIScan-FL assay to track and quantitatively analyze cell motility. The results revealed that most fatty acids suppressed both the distance and speed of cell motility, with crotonic acid displaying the most significant inhibitory effect (Fig. 1, G to J). Based on these observations, we found that, among the tested fatty acids, crotonic acid markedly suppressed cell migration and motility, whereas butyric acid notably inhibited cell proliferation (Fig. 1K).

Given that previous studies have well-established the link between butyric acid and cancer growth, we focused our investigation on the role of crotonic acid in cancer progression. By treating breast cancer cells with varying concentrations of crotonate, we found that a low concentration (2.5 mM) effectively suppressed cell migration (Fig. 1L), whereas a higher concentration (15 mM) was required to inhibit cell proliferation (Fig. 1M and fig. S1C). Collectively, these data indicate that crotonate is a potent suppressor of breast cancer cell migration and motility.

### Crotonyl-CoA catalyzed by ACSS2 inhibits cancer cell migration

**Fig. 2.**
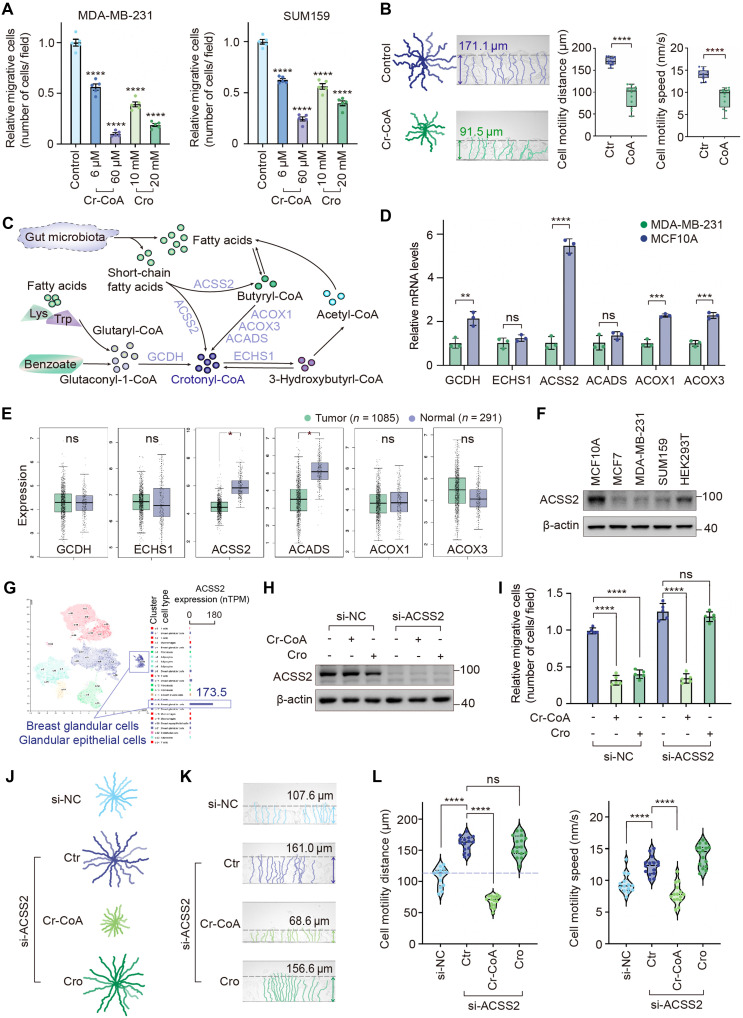
Crotonyl-CoA generated by ACSS2 inhibits cancer cell migration. (**A**) Transwell assays in crotonate (Cro)– and crotonyl-CoA (Cr-CoA)–treated (72 hours) breast cancer cells. Data represent means ± SD. ****P < 0.0001, unpaired *t* test with Welch’s correction. (**B**) The movement trajectories of Cr-CoA (60 μM, 72 hours)–treated SUM159 cells and the statistical distance and speed of cell motility. ****P < 0.0001, unpaired *t* test with Welch’s correction. (**C**) Diagram of the metabolic patterns of crotonate and Cr-CoA. (**D**) The mRNA levels of acyl-CoA synthetases detected by quantitative real-time polymerase chain reaction (qPCR). Data represent means ± SD. **P < 0.01; ***P < 0.001; ****P < 0.0001, unpaired *t* test with Welch’s correction. (**E**) The mRNA expression of these acyl-CoA synthetases in The Cancer Genome Atlas (TCGA) database. *P < 0.05, unpaired *t* test with Welch’s correction. (**F**) Western blot (WB) analysis of ACSS2 in different cell lines. (**G**) The single-cell mRNA expression profile of ACSS2 in breast tissue on the protein atlas website. nTPM, normalized TPM. (**H**) The efficacy of ACSS2 small interfering RNAs (siRNAs). (**I** to **L**) ACSS2–knocked down SUM159 cells were treated by Cr-CoA (30 μM, 72 hours) or crotonate (20 mM, 72 hours) for transwell (I) and TAXIScan-FL (J to L) assays. The trajectory curves were plotted (J) and the motility distance and speed were measured (K and L). Data represent means ± SD (I). ****P < 0.0001, unpaired *t* test with Welch’s correction.

To further investigate whether crotonyl-CoA mediates the suppressive effects of crotonate on cell migration, we treated breast cancer cells with crotonyl-CoA at varying concentrations. The results demonstrated that crotonyl-CoA markedly inhibited the migration of MDA-MB-231 and SUM159 cells at a lower concentration than crotonate (Fig. 2A). Notably, crotonyl-CoA treatment significantly reduced both the distance and speed of cell motility (Fig. 2B). These findings indicate that crotonyl-CoA exerts a more potent inhibitory effect on cancer cell migration compared to crotonate.

Crotonyl-CoA can be synthesized from crotonate by ACSS2 (*25*). In addition, other acyl-CoAs, such as butyryl-CoA, 3-hydroxybutyryl-CoA, and glutaryl-CoA, can also be transformed into crotonyl-CoA through acyl-CoA synthetases including ACOX1, ACOX3, ACADS, ECHS1, and GCDH (Fig. 2C). To identify the key acyl-CoA synthetases responsible for crotonyl-CoA synthesis in breast cancer, we first measured the mRNA levels of six enzymes in both the breast epithelial cell line MCF10A and the breast cancer cell line MDA-MB-231. Results showed that four acyl-CoA synthetases—GCDH, ACSS2, ACOX1, and ACOX3—were significantly down-regulated in breast cancer cells, with ACSS2 showing the most pronounced reduction (Fig. 2D). In addition, database analysis revealed that among the six enzymes, both ACSS2 and ACADS were down-regulated in cancer tissues (Fig. 2E), and low ACSS2 expression was associated with poor outcomes in breast cancer patients (fig. S2A). We further confirmed that ACSS2 was predominantly expressed in breast epithelial cells, as shown by Western blot (WB) in various cell lines (Fig. 2F) and mouse mammary tissues (fig. S2B) and by single-cell analysis from the Protein Atlas database (Fig. 2G). Based on these findings, ACSS2 may act as a tumor suppressor in breast cancer and is proposed as a candidate enzyme responsible for mediating the inhibitory effect of crotonate.

Next, we evaluated the functional role of ACSS2 and found that ACSS2 overexpression suppressed breast cancer cell migration (fig. S2, C and D). Moreover, we examined whether ACSS2 knockdown affects the suppressive effect of crotonate or crotonyl-CoA. Results showed that ACSS2 knockdown attenuated the inhibitory effect of crotonate on cell migration, whereas crotonyl-CoA retained its inhibitory role (Fig. 2, H and I). Similarly, analysis of cell motility tracks revealed that ACSS2 knockdown increased both the distance and speed of cell movement. Notably, crotonate lost its ability to inhibit cell motility in ACSS2-deficient cells, while crotonyl-CoA remained effective (Fig. 2, J to L). Together, these findings demonstrate that the conversion of crotonate to crotonyl-CoA by ACSS2 is essential for the suppressive effect of crotonate on cancer cell migration.

### Crotonate reduces genome-wide occupancy of H3K27 methylation

**Fig. 3.**
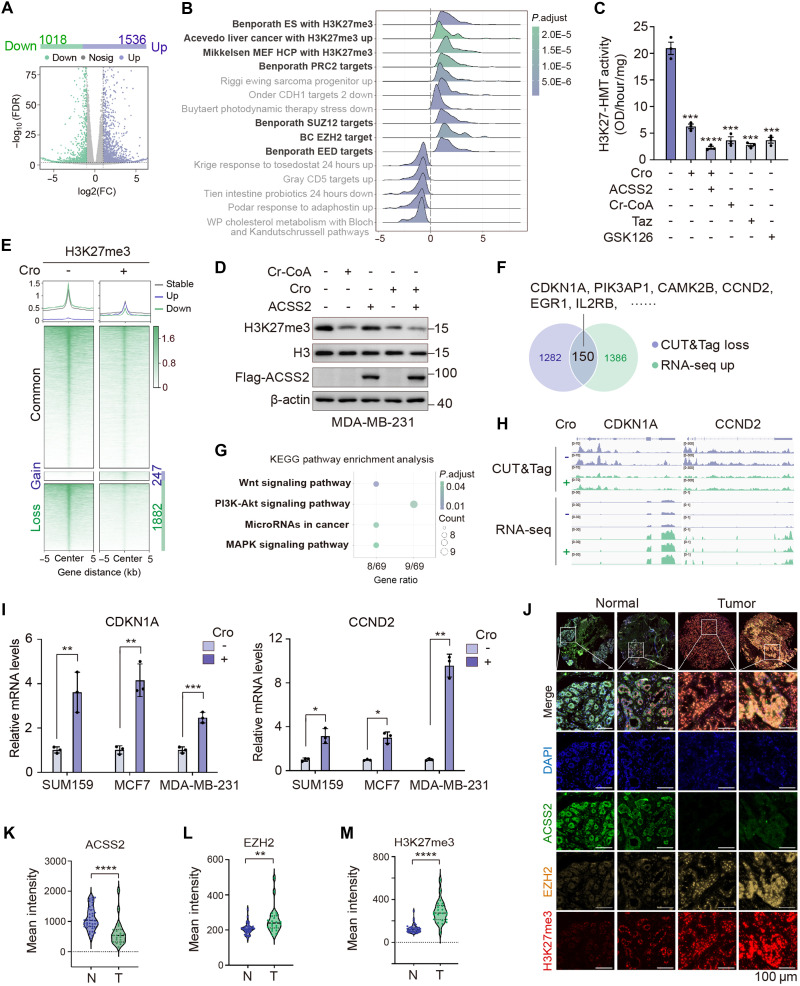
Crotonate decreases H3K27me3 occupancy. (**A**) Volcano plots showing the transcript levels of crotonate-treated versus untreated cells (*P* < 0.05, fold change > 2.0). (**B**) GSEA showing the top 15 signaling pathways. (**C**) MDA-MB-231 cells were treated respectively with crotonate (20 mM) (with or without ACSS2 overexpression), Cr-CoA (60 μM), tazemetostat (10 μM), and GSK126 (10 μM) for 72 hours. Nuclear proteins were extracted for detecting the H3K27 methyltransferase activity. Data represent the means ± SD. ***P < 0.001; ****P < 0.0001, unpaired *t* test with Welch’s correction. (**D**) WB analysis of H3K27me3 in Cr-CoA (60 μM)– and crotonate (20 mM, 72 hours)–treated MDA-MB-231 cells with or without ACSS2 overexpression. (**E**) Heatmaps and profiles of H3K27me3 CUT&Tag signals sorted based on differential H3K27me3 peaks. (**F**) The genes with decreased enrichment obtained from H3K27me3 CUT&Tag were overlapped with the up-regulated genes from RNA-seq (*P* < 2.22 × 10^−16^). (**G**) KEGG pathway enrichment analysis on the overlapped 150 genes. (**H**) CUT&Tag and RNA-seq genomic tracks of representative genes. (**I**) mRNA levels of CDKN1A and CCND2 in crotonate (20 mM, 72 hours)–treated breast cancer cells by qPCR. Data represent means ± SD. *P < 0.05; **P < 0.01; ***P < 0.001, unpaired *t* test with Welch’s correction. (**J**) Multiple immunofluorescence staining in breast cancer tissues (*n* = 22) and adjacent normal tissues (*n* = 40) using ACSS2, EZH2, and H3K27me3 antibodies. DAPI, 4′,6-diamidino-2-phenylindole. (**K** to **M**) The fluorescence intensities of EZH2, H3K27me3, and ACSS2. **P < 0.01; ****P < 0.0001, unpaired *t* test with Welch’s correction. N, normal; T, tumor.

To elucidate the mechanism by which crotonate inhibits breast cancer cell migration, we conducted RNA sequencing (RNA-seq) analysis on crotonate-treated MDA-MB-231 cells and identified 1536 up-regulated and 1018 down-regulated genes (Fig. 3A). Gene set enrichment analysis (GSEA) revealed that a substantial proportion of these up-regulated genes were enriched in pathways related to histone H3K27me3 and PRC2 complex (Fig. 3B), suggesting a potential association between crotonate treatment and the regulation of H3K27me3. To test this hypothesis, we assessed H3K27 methyltransferase activity in MDA-MB-231 cells. The results demonstrated that crotonate and crotonyl-CoA significantly suppressed H3K27 methyltransferase activity, comparable to the effects of two EZH2 inhibitors, tazemetostat and GSK126 (Fig. 3C). ACSS2 overexpression enhanced the inhibitory effect of crotonate (Fig. 3C). We also observed decreased levels of H3K27me3 in MDA-MB-231 cells treated with crotonate or crotonyl-CoA (Fig. 3D), suggesting that H3K27me3 may be involved in the gene regulation induced by crotonate.

Next, CUT&Tag assays were performed to assess changes in H3K27me3 occupancy following crotonate treatment. Results revealed a global reduction in H3K27me3 occupancy in crotonate-treated cells compared to control cells (Fig. 3E). By integrating the genes exhibiting down-regulated H3K27me3 peaks identified through CUT&Tag with the up-regulated gene expression data obtained from RNA-seq, we identified 150 target genes that were up-regulated as a result of reduced H3K27me3 occupancy induced by crotonate (Fig. 3F). Kyoto Encyclopedia of Genes and Genomes (KEGG) pathway enrichment analysis revealed that these genes were mainly enriched in pathways related to Wnt, phosphatidylinositol 3-kinase (PI3K)–Akt, and mitogen-activated protein kinase (MAPK) signaling (Fig. 3G), which play critical roles in cancer progression. The genomic tracks of representative genes, such as CDKN1A, are illustrated (Fig. 3H and fig. S3A), and their expression levels were validated in cancer cell lines (Fig. 3I and fig. S3B). These findings indicate that EZH2-mediated H3K27me3 occupancy is involved in the regulation of gene expression induced by crotonate treatment.

To further confirm the association between crotonate and EZH2-mediated H3K27me3 regulation, we examined the expression levels of ACSS2, EZH2, and H3K27me3 in breast cancer and adjacent normal tissues using immunofluorescence (Fig. 3J). Both EZH2 and H3K27me3 levels were elevated in cancer tissues compared to normal tissues. In contrast, ACSS2 expression was reduced in cancer tissues relative to normal tissues (Fig. 3, K to M). A negative correlation was observed between EZH2/H3K27me3 levels and ACSS2 level (fig. S3, C to F), suggesting a potential inverse relationship between crotonyl-CoA and EZH2 activity.

### Crotonate promotes proteasomal degradation of EZH2

**Fig. 4.**
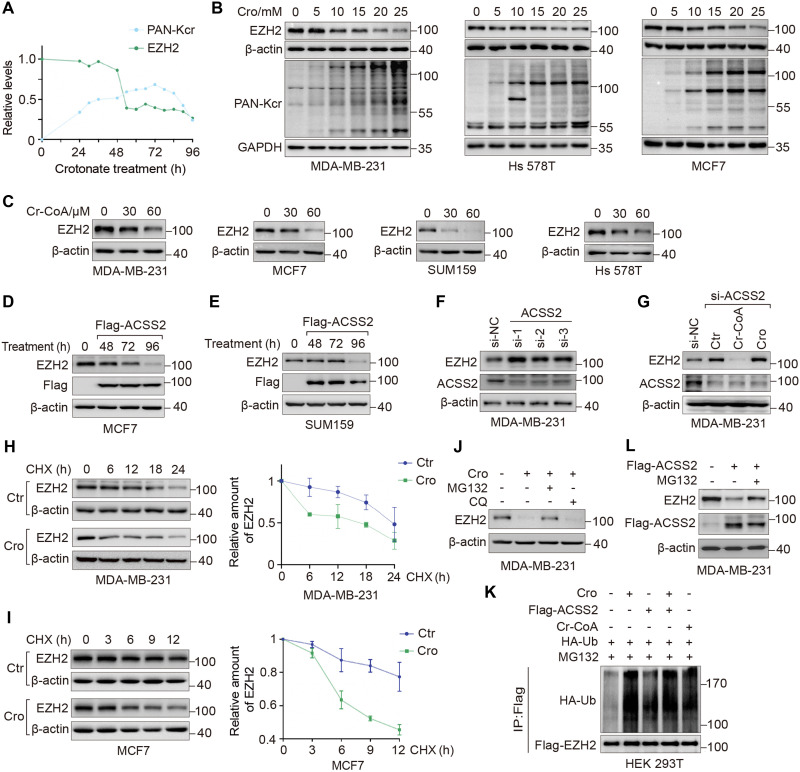
Crotonate promotes EZH2 degradation. (**A**) The relative abundances of EZH2 and PAN-Kcr were quantified in crotonate (20 mM)–treated MDA-MB-231 cells. (**B**) WB analysis of EZH2 and PAN-Kcr in different breast cancer cells treated by crotonate (72 hours). (**C**) WB analysis of EZH2 levels in different breast cancer cells treated by Cr-CoA (72 hours). (**D** and **E**) WB analysis of EZH2 levels in ACSS2-overexpressing cells. (**F**) WB analysis of EZH2 levels in ACSS2 siRNA–treated MDA-MB-231 cells. NC, negative control. (**G**) ACSS2 siRNA–treated MDA-MB-231 cells were treated with Cr-CoA (30 μM) or crotonate (20 mM) for 72 hours, followed by WB. (**H** and **I**) MDA-MB-231 and MCF7 cells were treated with crotonate (20 mM, 48 hours), together with CHX at 100 μg/ml for the indicated times. The protein levels of EZH2 were normalized to β-actin level at different time points from three independent experiments. (**J**) After treating MDA-MB-231 cells with crotonate (20 mM, 60 hours), cells were treated by MG132 (25 μM, 12 hours) or CQ (25 μM, 12 hours), followed by WB. (**K**) Hemagglutinin (HA)–ubiquitin (Ub) and Flag-EZH2 were cotransfected into HEK293T cells. Flag-EZH2 ubiquitination was detected by coimmunoprecipitation (Co-IP). (**L**) WB analysis of EZH2 levels in ACSS2-overexpressed cells with MG132 (25 μM, 12 hours) treatment. h, hours.

To investigate whether crotonate/crotonyl-CoA regulates EZH2 level, we first monitored the temporal dynamics of EZH2 and total crotonylation levels following crotonate treatment. The results showed that EZH2 protein levels were markedly reduced 48 hours after crotonate treatment, concomitant with an increase in total crotonylation levels (Fig. 4A and fig. S4A). Furthermore, the protein level of EZH2 was decreased in a dose-dependent manner in multiple breast cancer cell lines upon crotonate treatment (Fig. 4B), whereas mRNA levels remained unchanged (fig. S4B). We further investigated the impact of crotonyl-CoA on EZH2 levels in breast cancer cells. Consistent with the effects of crotonate, crotonyl-CoA also reduced EZH2 protein levels in multiple breast cancer cell lines (Fig. 4C).

Given that ACSS2 catalyzes the conversion of crotonate to crotonyl-CoA, we examined the regulation of ACSS2 on EZH2 protein level. Results demonstrated that overexpression of ACSS2 notably decreased EZH2 levels in breast cancer cells (Fig. 4, D and E), whereas ACSS2 knockdown resulted in a marked increase in EZH2 levels (Fig. 4F). Notably, the increase of EZH2 levels induced by ACSS2 knockdown was reversed by crotonyl-CoA treatment, whereas crotonate had no such effect (Fig. 4G), suggesting that ACSS2 regulates EZH2 through the production of crotonyl-CoA. These findings demonstrate that crotonate down-regulates EZH2 level via ACSS2-mediated conversion of crotonate to crotonyl-CoA.

As mentioned above, crotonate down-regulates EZH2 protein levels without affecting its mRNA levels; therefore, we speculated that crotonate may regulate EZH2 protein stability. A cycloheximide (CHX) chase assay revealed that crotonate treatment shortened the half-life of EZH2 (Fig. 4, H and I), indicating that crotonate reduces EZH2 stability.

Moreover, cells were treated with crotonate in combination with either the proteasomal inhibitor *N*-carbobenzyloxy-l-leucyl-l-leucyl-l-leucinal (MG132) or the lysosomal inhibitor chloroquine (CQ). Results showed that MG132 treatment blocked the down-regulation of EZH2 protein levels induced by crotonate, while CQ treatment had no obvious effect (Fig. 4J), suggesting that crotonate regulates EZH2 degradation via the proteasome pathway. In addition, crotonate treatment also increased the polyubiquitination of EZH2 (Fig. 4K). Furthermore, we observed that the down-regulation of EZH2 caused by ACSS2 overexpression could be restored by MG132 treatment (Fig. 4L), suggesting the regulation of ACSS2 on EZH2 degradation. Collectively, these data demonstrate that crotonate promotes ubiquitin-dependent proteasomal degradation of EZH2.

### EZH2 crotonylation induced by crotonate inhibits cell migration

**Fig. 5.**
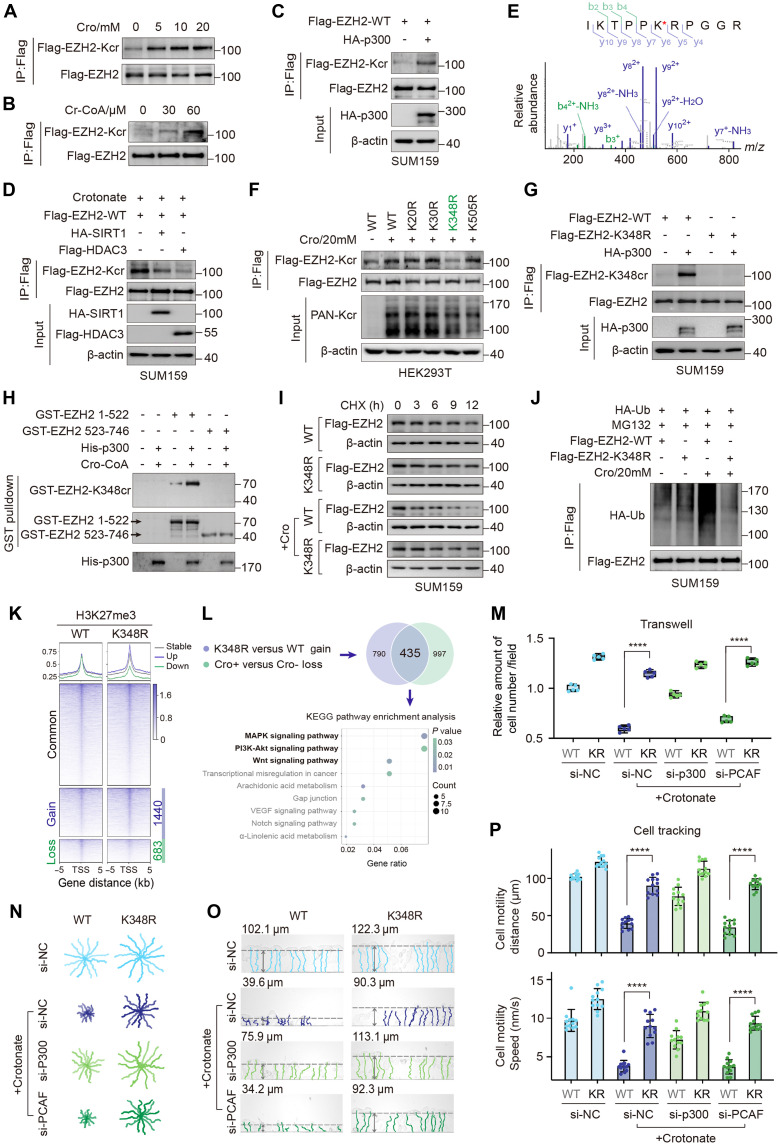
EZH2 crotonylation inhibits cell migration. (**A** and **B**) Flag-EZH2–overexpressed HEK293T cells were treated with crotonate (A) and Cr-CoA (B) at different doses, followed by Co-IP using anti-Flag antibody to detect Flag-EZH2-Kcr. (**C**) WB analysis of Flag-EZH2-Kcr level in p300-overexpressed SUM159 cells. (**D**) WB analysis of Flag-EZH2-Kcr level in Sirt1 and HDAC3-overexpressed SUM159 cells. (**E**) Crotonylation sites of EZH2 were analyzed by mass spectrometry. *m*/*z*, mass/charge ratio. (**F**) Co-IP assays to detect Flag-EZH2-Kcr level of Flag-EZH2-WT and mutants. (**G**) Co-IP assays to detect Flag-EZH2-K348cr level in p300-overexpressed SUM159 cells using specifically recognizing EZH2-K348cr antibody. (**H**) In vitro crotonylation assay. GST, glutathione *S*-transferase. (**I**) The half-life of Flag-EZH2-WT and K348R mutant in SUM159 cells treated with or without crotonate (20 mM, 48 hours), together with CHX (100 μg/ml). (**J**) HA-Ub, Flag-EZH2-WT, and K348R were transfected into stable SUM159-shEZH2 cells. After 12 hours, cells were treated with crotonate (20 mM, 48 hours), together with MG132 (25 μM, 12 hours), followed by Co-IP to detect the ubiquitination of Flag-EZH2. (**K**) Heatmaps and profiles of H3K27me3 CUT&Tag signals on based on the differential peaks between EZH2-WT and K348R stable cells treated by crotonate. TSS, transcript start sites. (**L**) Overlap analysis of regulated genes between the EZH2-K348R versus WT CUT&Tag dataset (Fig. 5K) and the crotonate-treated versus untreated dataset (Fig. 3E). (**M** to **P**) Transwell (M) and TAXIScan-FL (N to P) assays in control, p300, and PCAF siRNA–treated SUM159 cells. The trajectory curves were plotted (N and O), and the distance and speed (P) of single cell movement were measured. Data represent the means ± SD. ****P < 0.0001, unpaired *t* test with Welch’s correction. KR, Lys (K) mutated to Arg (R).

Given that crotonate induces nonhistone protein crotonylation, we examined whether EZH2 can be modified by crotonylation. Our results demonstrated that both crotonate and crotonyl-CoA effectively induced crotonylation of EZH2 (EZH2-Kcr) (Fig. 5, A and B). Endogenous EZH2-Kcr was detectable in cells treated with decrotonylase inhibitors (fig. S5A). Next, we investigated the crotonyltransferases responsible for EZH2-Kcr by cotransfecting Flag-EZH2 with a panel of acetyltransferases, including human MOF (hMOF), Tip60, p300, CBP, and PCAF. We found that EZH2 was strongly crotonylated by p300 and moderately crotonylated by CBP, whereas it was acetylated by CBP and PCAF (fig. S5B). We further confirmed the interaction between p300 and EZH2 (fig. S5C) and the regulation of p300 on EZH2-Kcr levels through gain- and loss-of-function assays (Fig. 5C and fig. S5D). Because both HDACs and sirtuins (SIRTs) are reported to function as decrotonylases for nonhistone proteins, we treated cells with trichostatin A, an HDAC inhibitor, or nicotinamide, a SIRT inhibitor. We observed that both treatments increased EZH2-Kcr levels (fig. S5E), indicating that both HDACs and SIRTs are involved in the decrotonylation of EZH2. Further analysis revealed that SIRT1 and HDAC3 were the decrotonylases involved in this process (Fig. 5D and fig. S5, F to J). These findings demonstrate that p300 and SIRT1/HDAC3 act as the writer and eraser for EZH2 crotonylation.

To identify the specific lysine residues in EZH2 that undergo crotonylation in vivo, we transfected cells with FLAG-EZH2 and treated them with crotonate. Mass spectrometry analysis identified four lysine residues (K505, K348, K30, and K20) of EZH2 as potential crotonylation sites (Fig. 5E). We generated four mutants in which each lysine residue was substituted with arginine and then transfected the different EZH2 mutants into crotonate-treated cells. The results showed that only the K348R mutant substantially reduced the level of EZH2-Kcr (Fig. 5F). Notably, K348 is an evolutionarily conserved residue in EZH2 across species (fig. S5K). To precisely evaluate the crotonylation of EZH2 at K348 (EZH2-K348cr), we developed a rabbit polyclonal antibody specifically recognizing crotonylated EZH2 at K348 (fig. S5L). We found that p300 could catalyze the K348cr of wild-type (WT) EZH2 but not the K348R mutant (Fig. 5G). Furthermore, we performed the in vitro crotonylation assay using recombinant His-p300, EZH2 substrate, and crotonyl-CoA. Results showed that p300 effectively catalyzes the K348cr of EZH2 in vitro (Fig. 5H). Collectively, these findings demonstrate that p300 crotonylates EZH2 at the K348 residue.

We further investigated the relationship between EZH2-K348cr and its degradation. Upon crotonate treatment, the half-life of WT EZH2 was significantly shortened, whereas that of the crotonylation-deficient K348R mutant remained unchanged (Fig. 5I and fig. S5, M to O). Moreover, crotonate treatment promoted ubiquitination of WT EZH2, but not of the K348R mutant (Fig. 5J). Consistently, overexpression of the E3 ligase TRAF6 increased ubiquitination of WT EZH2, but not that of the K348R mutant (fig. S5P). These results demonstrate that crotonylation of EZH2 at K348 triggers TRAF6-mediated ubiquitination and degradation of EZH2.

Furthermore, stable cell lines expressing either WT or K348R EZH2 were established (fig. S6A) and used to compare global H3K27me3 occupancy. CUT&Tag assays revealed that cells expressing the K348R mutant exhibited a global increase in H3K27me3 occupancy compared to cells expressing WT EZH2 upon crotonate treatment (Fig. 5K). The EZH2-K348R/WT CUT&Tag dataset (Fig. 5K) was integrated with the crotonate-treated/untreated CUT&Tag dataset (Fig. 3E). A total of 435 genes were identified as coregulated by EZH2-K348cr and crotonate treatment, and these genes are predominantly enriched in the MAPK, PI3K-Akt, and Wnt signaling pathways (Fig. 5L). We selected the representative genes to show their genomic tracks, including the MAPK signaling–related genes EFNA3 and FGF19, as well as the Wnt signaling-related genes WNT10A and CCND2 (fig. S6B). Their mRNA levels were also validated in EZH2-WT and K348R cells (fig. S6C). These results suggest that EZH2 K348cr reduces H3K27me3 occupancy.

Moreover, the effect of EZH2-K348cr on cancer cell migration was evaluated. Cells expressing the EZH2 K348R mutant exhibited enhanced migratory capacity compared to those expressing WT EZH2 upon crotonate treatment (fig. S6D). To further clarify the role of EZH2-K348cr, we knocked down p300 or PCAF in crotonate-treated WT and K348R cells. The results showed that p300 knockdown largely reversed the inhibitory effect of crotonate, whereas PCAF knockdown did not result in obvious changes (Fig. 5, M to P, and fig. S6E). These findings indicate that p300-induced EZH2-K348cr inhibits cancer cell migration.

### Both crotonate and crotonyl-CoA effectively inhibits breast cancer cell metastasis in vivo

**Fig. 6.**
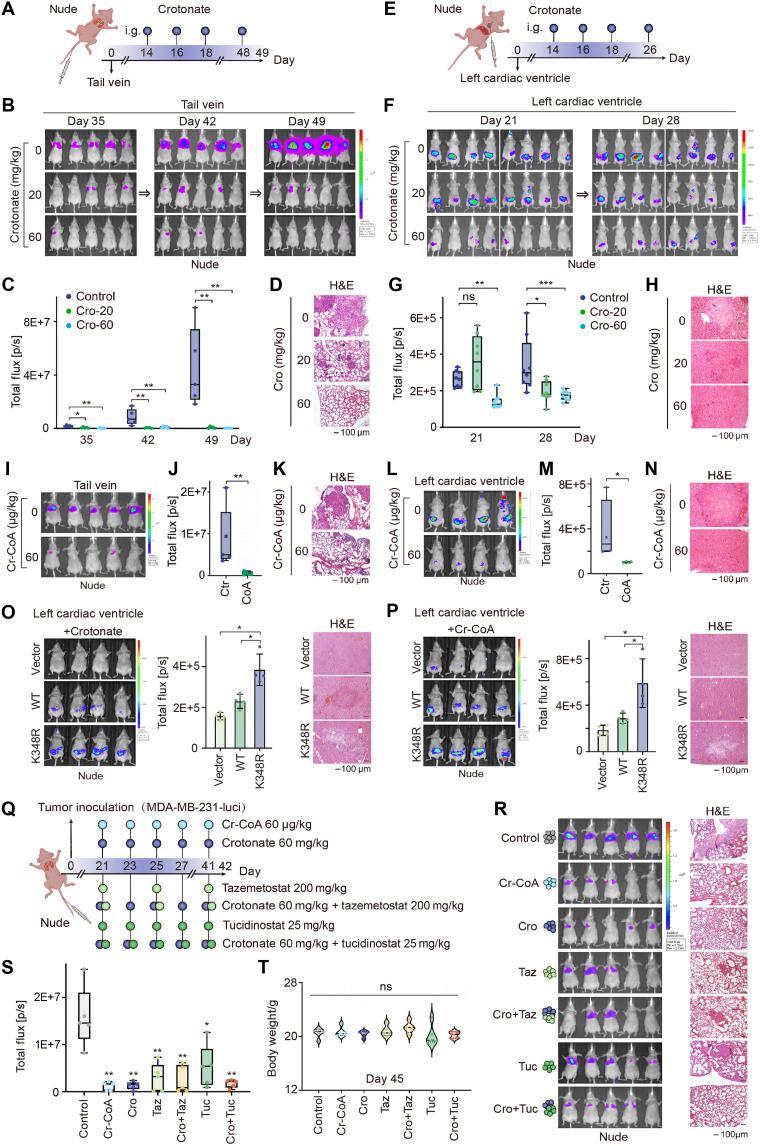
Crotonate/crotonyl-CoA inhibits breast cancer cell metastasis in vivo. (**A** to **D**) MDA-MB-231-luci cells were injected into nude mice through tail vein. After 14 days, mice were divided into three groups (*n* = 5 per group) and treated with saline or crotonate by gavage [intragastric (i.g.)] every 2 days (A). IVIS imaging on days 35, 42, and 49 (B), the fluorescence intensity (C), and H&E staining in the lung (D). (**E** to **H**) MDA-MB-231-luci cells were injected into nude mice via intracardiac injection. Fourteen days later, the mice were divided into three groups (*n* = 8 per group) and treated with saline or crotonate via gavage every 2 days (E). IVIS imaging on days 21 and 28 (F), fluorescence intensity (G), and H&E staining of the liver tissues (H). (**I** to **K**) MDA-MB-231-luci cells were injected into nude mice through the tail vein. Fourteen days later, mice were divided into two groups (*n* = 5 per group) and treated with saline or Cr-CoA by gavage every 2 days. (**L** to **N**) MDA-MB-231-luci cells were injected into nude mice through heart injection. Mice were divided into two groups (*n* = 4 per group) and treated with saline or Cr-CoA by gavage every 2 days. (**O** and **P**) MDA-MB-231-luci cells stably overexpressing EZH2-WT or K348R were inoculated into the left cardiac ventricle, and the mice were given crotonate (60 mg/kg) (O) or Cr-CoA (60 μg/mg) (P) by gavage every 2 days. (**Q** to **T**) MDA-MB-231-luci cells were injected into nude mice through tail vein. Mice were divided into seven groups (*n* = 5 per group) and administrated with the indicated drugs by gavage for 3 weeks. Data represent the means ± SD. *P < 0.05; **P < 0.01; ***P < 0.001, unpaired *t* test with Welch’s correction (C, G, J, M, O, P, S, and T).

Given the suppressive role of crotonate on cell migration, we evaluated its potential therapeutic efficacy in vivo. We injected MDA-MB-231-Luc-D3H2LN cells intravenously into nude mice and administrated crotonate at 14 days postinjection by oral gavage at doses of 20 and 60 mg/kg (Fig. 6A). To monitor cancer cell metastasis, we conducted bioluminescence imaging at different time points. Results showed that crotonate treatment markedly suppressed lung metastasis of cancer cells starting from 35 days postinjection, and this suppressive effect persisted until 49 days postinjection (Fig. 6, B and C). Furthermore, hematoxylin and eosin (H&E) staining confirmed the presence of lung metastases (Fig. 6D). Notably, no significant differences in body weight were observed between the crotonate-treated and control groups (fig. S7A), indicating that crotonate exhibited low toxicity at the tested doses. In addition, we inoculated MDA-MB-231-Luc-D3H2LN cells intracardially into nude mice, and bioluminescence imaging demonstrated that crotonate administration markedly suppressed liver metastasis of cancer cells, as supported by H&E staining of liver tissues (Fig. 6, E to H, and fig. S7B). Moreover, to investigate whether crotonate retains its inhibitory effect on cancer cell metastasis in immunocompetent mice, we evaluated its impact in C57 mice. Results indicated that crotonate suppressed lung and liver metastasis of cancer cells in C57 mice, although a higher dose (200 mg/kg) was required (fig. S7, C to J). Collectively, these findings demonstrate the potent inhibitory effect of crotonate on cancer cell metastasis in both immunodeficient and immunocompetent mouse models.

Since crotonyl-CoA mediates the suppressive effects of crotonate on cell migration, we further investigated its role in mouse models. Results showed that crotonyl-CoA effectively inhibited lung and liver metastasis of breast cancer cells at a relatively low dose (60 μg/kg), and H&E staining confirmed the presence of metastases (Fig. 6, I to N, and fig. S7, K and L).

To investigate whether EZH2 crotonylation plays a critical role in crotonate-mediated metastasis regulation, we used MDA-MB-231-Luc-D3H2LN cells stably expressing EZH2-WT or K348R in animal experiments. Fourteen days after intracardial inoculation of the cells into mice, the animals were administered either crotonate or crotonyl-CoA. The data showed that both crotonate and crotonyl-CoA significantly inhibited liver metastasis in cells expressing EZH2-WT, compared to those expressing EZH2-K348R (Fig. 6, O and P, and fig. S7M). These findings indicate that EZH2-K348cr largely mediates the inhibitory effect of crotonate on cancer cell metastasis.

To further evaluate the efficacy of crotonate and crotonyl-CoA, we compared their suppressive effects on metastasis with some epigenetic inhibitors (Fig. 6Q). Tazemetostat is a first-in-class Food and Drug Administration (FDA)–approved oral EZH2 inhibitor for follicular lymphoma and epithelioid sarcoma. Tucidinostat, a clinically approved HDAC inhibitor, is used for treating T cell leukemia and lymphoma. Results indicated that both crotonate and crotonyl-CoA individually exhibited a more pronounced antimetastatic effect compared to tazemetostat or tucidinostat alone (Fig. 6, R to T). However, combining crotonate or crotonyl-CoA with these inhibitors did not yield a synergistic antimetastatic effect. Collectively, these findings highlight the robust antimetastatic potential of crotonate and crotonyl-CoA.

### Combination of crotonate administration and immunotherapy elicits a synergistic anticancer effect

**Fig. 7.**
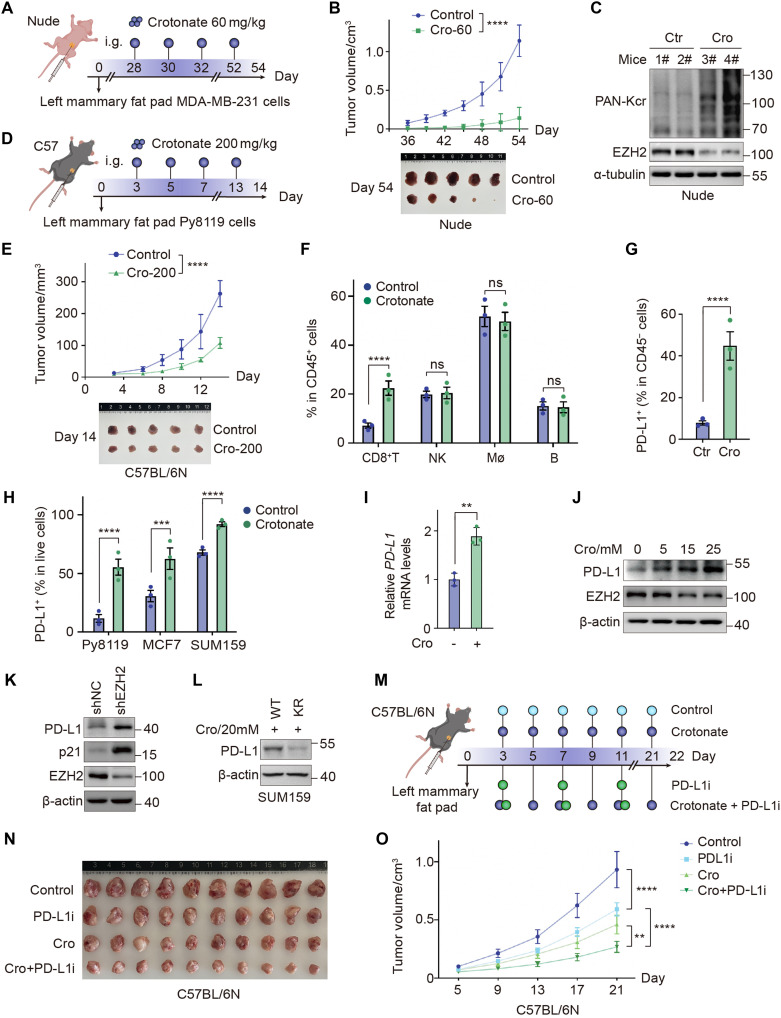
Combination of crotonate and anti-PD-L1 suppress breast cancer progression. (**A** to **C**) MDA-MB-231 cells were injected into the mammary fat pad of nude mice (A). Mice were divided into two groups and given saline or crotonate (60 mg/kg) by gavage every 2 days. Tumor growth curve and image of primary tumors (B). Total protein was extracted from the tumor tissues, followed by WB (C). (**D** to **G**) Py8119 cells were injected into C57BL/6N mice (D). Mice were divided into 2 groups and given saline or crotonate (200 mg/kg) by gavage every 2 days. Tumor growth curve and image of primary tumors (E). Flow cytometry analysis of the tumor tissues (F and G). ****P < 0.0001, two-way analysis of variance (ANOVA) (B and E) or unpaired *t* test with Welch’s correction (F and G). (**H**) Flow cytometry analysis of breast cancer cells treated with crotonate (20 mM, 72 hours). Data represent the means ± SEM. ***P < 0.001; ****P < 0.0001, unpaired *t* test with Welch’s correction. (**I**) qPCR detecting the mRNA level of PD-L1 in crotonate (20 mM, 72 hours)–treated MDA-MB-231 cells. Data represent the means ± SD. **P < 0.01, unpaired *t* test with Welch’s correction. (**J**) WB analysis of PD-L1 level in crotonate-treated MDA-MB-231 cells. (**K**) WB analysis of PD-L1 level in SUM159-shEZH2 and control stable cells. (**L**) WB analysis of PD-L1 level in SUM159-EZH2-WT and K348R stable cells. (**M** to **O**) Py8119 cells were injected into C57BL/6N mice. Mice were divided into four groups (*n* = 10 per group) and administrated with crotonate and/or InVivoMAb anti-PD-L1 (M). Tumor growth curve (N) and image of primary tumors (O) were shown. Data represent the means ± SD. **P < 0.01; ****P < 0.0001, two-way ANOVA.

Given that crotonate also inhibits cancer cell proliferation in vitro, we further investigated its effects on tumor growth in vivo (Fig. 7A). Administration of crotonate at 60 mg/kg significantly suppressed orthotopic mammary tumor growth in nude mice (Fig. 7B and fig. S8A). By extracting and analyzing proteins from tumor tissues, we demonstrated that crotonate-treated tumors exhibited elevated global crotonylation levels and reduced EZH2 protein expression (Fig. 7C). These findings suggest that the inhibitory effect of crotonate on tumor growth is associated with crotonylation modification and EZH2 degradation.

Considering the association between EZH2 and immunosuppressive tumor microenvironments, we administered crotonate to immunocompetent mice for further investigation (Fig. 7D). Unlike the 60 mg/kg dose used in immunodeficient mice, a higher dose of 200 mg/kg was required to achieve comparable tumor growth inhibition in immunocompetent mice (Fig. 7E and fig. S8B). Immunofluorescence staining showed a decreased Ki67 level in crotonate-treated tumor tissues (fig. S8C). Notably, crotonate administration increased the intratumoral abundance of CD8^+^ T cells, whereas no significant increases were observed in other immune cell populations, including natural killer cells, macrophages, and B cells (Fig. 7F and fig. S8, D and E). Furthermore, crotonate administration markedly enhanced PD-L1 level in xenograft tumor tissues (Fig. 7G and fig. S8F). In addition, crotonate treatment also increased the PD-L1 levels in different breast cancer cell lines (Fig. 7H and fig. S8G).

To elucidate the detailed relationship between crotonate and PD-L1, we examined changes in PD-L1 levels following crotonate treatment in vitro. Our results confirmed that crotonate increased both mRNA and protein levels of PD-L1 (Fig. 7, I and J, and fig. S8H). In addition, EZH2 knockdown led to an increase in PD-L1 protein levels (Fig. 7K), suggesting a regulatory link between EZH2 and PD-L1. Moreover, overexpression of the EZH2 K348R mutant resulted in lower PD-L1 levels compared to WT EZH2 (Fig. 7L). These findings indicate that crotonylation-induced EZH2 degradation contributes to the up-regulation of PD-L1.

To enhance the efficacy of crotonate and overcome resistance to anti-PD-L1 therapy in mammary tumors, we treated immunocompetent mice bearing Py8119 cell–derived mammary tumors with crotonate alone, anti-PD-L1 antibody alone, or a combination of both (Fig. 7M). Results showed that the combination therapy significantly inhibited tumor growth compared to either monotherapy, exhibiting a synergistic anticancer effect (Fig. 7, N and O). Collectively, these results demonstrate that combination of crotonate and anti-PD-L1 antibody enhances responses of breast cancer cells to immunotherapy.

### Administration of crotonate maintains gut homeostasis

**Fig. 8.**
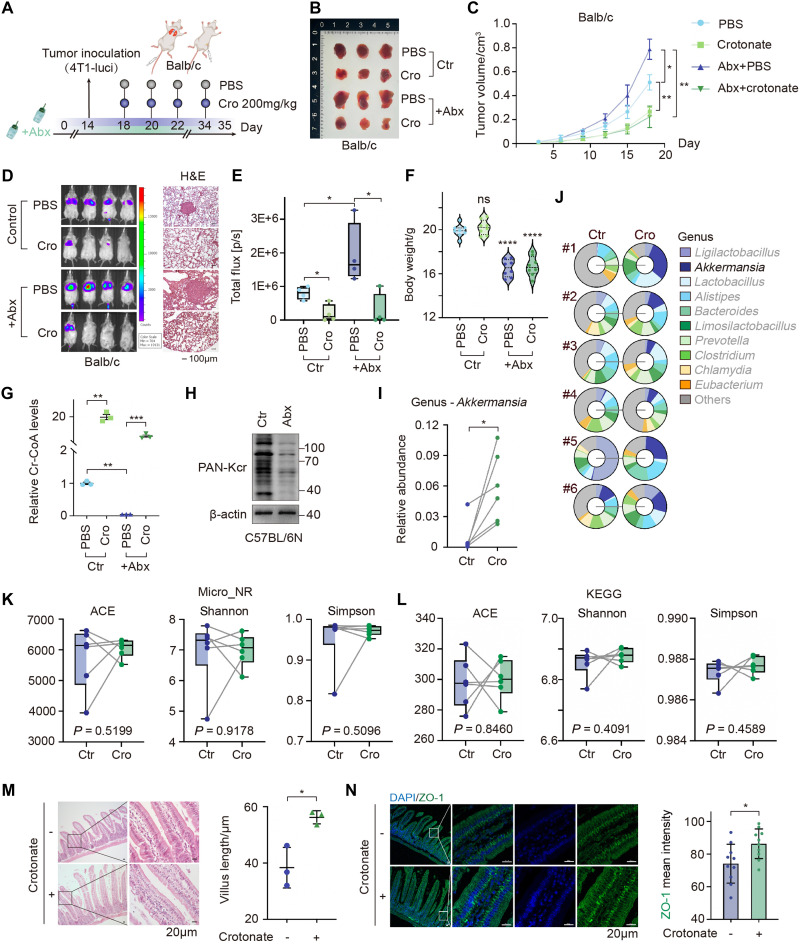
Oral administration of crotonate maintains gut homeostasis. (**A** to **C**) 4T1 cells were injected into the mammary fat pad of BALB/c mice. After mice were treated by antibiotics (Abx) for 2 weeks, mice were divided into four groups, and crotonate was administrated. Images of primary tumors and tumor growth curve (B and C). Data represent the means ± SD. *P < 0.05; **P < 0.01, two-way ANOVA. (**D** to **F**) 4T1-luci cells were injected into the tail vein of each group of mice. IVIS imaging, H&E staining of the lung tissues (D), the fluorescence intensity (E), and the mice weights (F). *P < 0.05; ****P < 0.0001, unpaired *t* test with Welch’s correction. (**G**) Cr-CoA was detected by high-performance liquid chromatography using the tissues from the xenograft tumors in (B). Data represent the means ± SD. **P < 0.01; ***P < 0.001, unpaired *t* test with Welch’s correction. (**H**) Total protein was extracted from the xenograft tumors in (B), followed by WB. (**I** to **L**) C57BL/6N mice were treated with crotonate by gavage once every 2 days for 3 weeks. Fecal samples were collected for metagenomic sequencing of intestinal microbes. The relative abundance of *Akkermansia* genus was shown (I and J). The microbial composition (K) and functional diversity (L) were evaluated using the ACE index, Shannon index, and Simpson index. (**M**) H&E staining showed the intestinal villi in mice (I), and the villus length was quantified. (**N**) Immunofluorescence staining was performed using the nuclei (DAPI, blue) and ZO-1 (Alexa Flour 488, green). Data represent the means ± SD. *P < 0.05, unpaired *t* test with Welch’s correction.

Crotonic acid is primarily derived from gut microbial metabolism. To investigate whether endogenous crotonic acid derived from gut microbial metabolism also inhibits tumor growth and metastasis, nude mice were treated with an antibiotic cocktail (Abx) to disrupt the gut microbiota. Subsequently, tumor cells were orthotopically inoculated into the mammary fat pad of mice following oral administration of crotonate (Fig. 8A). Results showed that Abx treatment alone promoted tumor growth compared to phosphate-buffered saline (PBS) treatment, suggesting that the normal gut microbiome exerts an anticancer effect. The supplementation of exogenous crotonate in germ-free mice significantly reduced tumor volumes (Fig. 8, B and C).

Furthermore, tumor cells were intravenously injected into Abx-treated mice. Abx treatment alone promoted lung metastasis of tumor cells and induced a significant reduction in body weight (Fig. 8, D to F, and fig. S9, A to C). However, oral administration of crotonate effectively reversed the Abx-induced lung metastases (Fig. 8, D to F). To further elucidate the potential mechanism underlying Abx-promoted tumor development, we measured the levels of crotonyl-CoA and global crotonylation modification in tumor cells. We observed that both crotonyl-CoA and crotonylation levels were markedly diminished in tumor tissues from Abx-treated mice, thereby providing a mechanistic explanation for the promotion of tumor growth by Abx (Fig. 8, G and H). Collectively, these results suggest that the disruption of gut microbiota led to a reduction in crotonic acid production, thereby promoting the growth and metastasis of tumor cells.

To further investigate whether oral administration of crotonate affects gut homeostasis by disturbing the gut microbiome, metagenomic analysis was conducted to examine the fecal microbiome of C57BL/6N mice treated with crotonate. We first assessed the composition of the gut microbiota and found that the relative abundance of *Akkermansia muciniphila* (Akk) was significantly higher in the crotonate-treated group compared with the control group (Fig. 8, I and J). In addition, the diversity of the gut microbiota was assessed using community richness and diversity indices, revealing no significant differences between the crotonate-treated group and the control group (Fig. 8, K and L). H&E staining of mouse intestine showed that the villus length was increased in crotonate-treated mice, suggesting a potential enhancing effect on intestinal mucosal structure and nutrient absorption capacity (Fig. 8M). To evaluate the function of the mucosal barrier, we examined the expression levels of several intestinal mucosal markers, including ZO-1 (absorptive cell marker), MUC2 (goblet cell marker), and CHGA (enteroendocrine cell marker). Results showed that the level of the tight junction protein ZO-1 was elevated in the intestinal mucosa of crotonate-treated mice (Fig. 8N), suggesting that crotonate may enhance the integrity of the mucosal barrier. In contrast, the levels of both MUC2 and CHGA remained largely unchanged (fig. S9D), indicating that crotonate may have no impact on mucus layer formation or intestinal inflammatory status. Collectively, these findings suggest that crotonate administration is beneficial for maintaining gut homeostasis and reinforcing mucosal barrier function.

## DISCUSSION

**Fig. 9.**
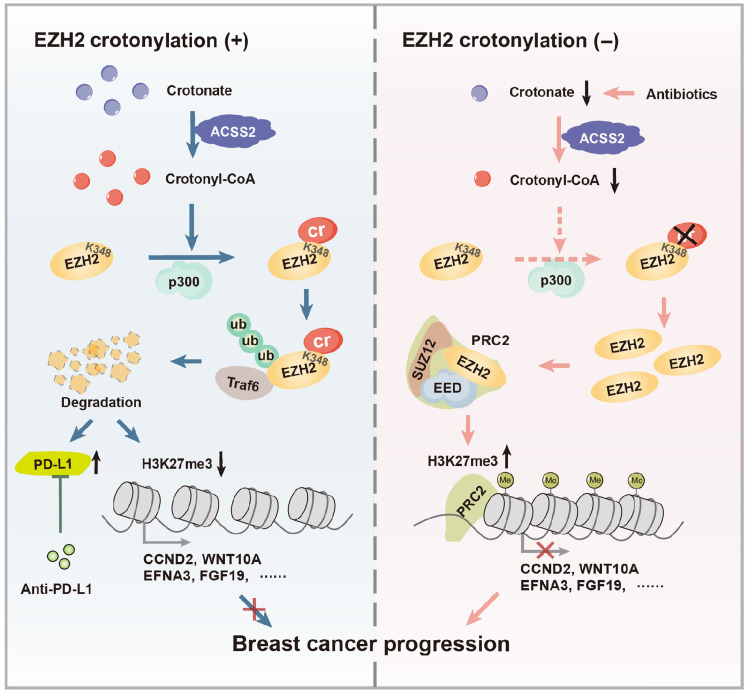
A working model.

In this study, we identified that SCFA crotonate promotes oncogenic EZH2 degradation through crotonylation at Lys^348^ of EZH2. Crotonate markedly inhibits breast cancer cell growth and metastasis, mediated by crotonyl-CoA. EZH2 crotonylation enhances the immunotherapy responses through up-regulating PD-L1 level. Our findings indicate that crotonyl-CoA, as the metabolic product of crotonate, plays an important role in blocking breast cancer progression and facilitating the immunotherapy responses (Fig. 9).

Distinct fatty acids can either promote or suppress tumor development depending on the specific biological and pathological context. For example, certain LCFAs, such as palmitic acid, oleic acid, and linoleic acid, have been shown to facilitate both primary tumor growth and distant metastasis in various types of cancer (*26*–*29*). In contrast, some MCFAs, such as decanoic acid and capric acid, decrease the viability of cancer cells, thereby exerting antitumor effects (*30*, *31*). Among the SCFAs, butyrate is the most well-characterized modulator of HDACs, playing a suppressive role in tumor growth. By systematically investigating the effects of distinct fatty acids on cell proliferation and migration in breast cancer, we observed that certain fatty acids inhibit both cell proliferation and migration, others promote both processes, while some selectively promote cell proliferation but inhibit cell migration (Fig. 1K). Notably, among these fatty acids, crotonate exhibits the most pronounced inhibitory effect on the migration of breast cancer cells. However, SCFAs are mixed products derived from gut microbiota metabolism, and whether the combination of crotonate and other SCFAs potentiates a synergistic anticancer effect remains to be investigated.

Dietary fibers derived from whole grains, fruits, and vegetables provide benefits to positively influencing multiple aspects of health. These fibers are fermented by gut microbiota into SCFAs, which play a pivotal role in suppressing inflammation and carcinogenesis in the gut and other organs (*32*). The concentrations of most SCFAs are highest in the colonic lumen and undetectable in peripheral blood (*33*). Only acetate reaches levels high enough to trigger GPR43-dependent signaling in peripheral tissues (*34*). There are no current reports quantifying crotonate levels in peripheral tissues due to limited studies. Analysis of The Cancer Genome Atlas (TCGA) database shows that SCFAs’ receptors GPR41 and GPR43 are overexpressed in breast cancer tissues, suggesting that crotonate may enter tumor cells through GPR41 or GPR43 recognition. Although we speculate that the amount of endogenous crotonate produced by gut bacteria may be insufficient to activate its receptors, oral administration of crotonate notably increases its concentration in the peripheral tissues. Both the crotonyl-CoA level and the global crotonylation level is elevated in mammary tumor tissues of mice following oral administration (Fig. 8, G and H), indirectly confirming that crotonate can reach tumor cells and exert its inhibitory function.

HATs catalyze various histone acylations, including acetylation, propionylation, butyrylation, and crotonylation, using a range of acyl-CoA cofactors. For most HATs, the preference for acyl-CoA is primarily determined by both chain length and corresponding concentrations of acyl-CoA (*35*–*37*). EZH2-K348 could be modified by PCAF-primed acetylation (*38*) or p300-mediated crotonylation (Fig. 5H). Crotonylation, rather than acetylation, at EZH2-K348 is primarily involved in the regulation of cell motility (Fig. 5, M to P). However, because p300 knockdown failed to fully rescue the crotonate-induced phenotype, the involvement of additional signaling pathways cannot be excluded. Acetylation at K348 enhances EZH2 stability (*38*), whereas crotonylation at K348 leads to its destabilization (Fig. 5C). These studies indicate that acetylation and crotonylation could occur at the same lysine residue (K348) in EZH2 and exert completely opposite functions. This provides a paradigm in nonhistone proteins that acetylation and crotonylation at the same lysine residue display contradictory biological functions. The distinct functions may be interpreted as different readers and cofactors recruited by acetylation or crotonylation separately, and this possibility is worthy of further investigation.

Dysregulation of EZH2 frequently occurs in various cancers, making targeting EZH2 a promising therapeutic strategy for breast cancer treatment. Although numerous EZH2 inhibitors have been developed for tumor treatment, their clinical efficacy in solid tumors remains limited. Therefore, combination therapy involving EZH2 inhibitors with immune checkpoint inhibitors, chemotherapy, or radiotherapy is considered a more effective approach. Here, we found that crotonate exhibits superior efficacy compared to the FDA-approved EZH2 inhibitor tazemetostat (Fig. 6R). One possible reason is that crotonate directly reduces EZH2 protein levels rather than solely inhibiting its methyltransferase activity. It is also possible that EZH2 is not the only downstream target of crotonate. Notably, the combination of crotonate and anti-PD-L1 significantly inhibited tumor growth compared to either monotherapy, demonstrating a synergistic anticancer effect (Fig. 7, N and O). Since both EZH2 inhibition and gut Akk abundance have been shown to enhance the efficacy of immunotherapy (*39*, *40*), these findings provide evidence that crotonate can enhance immunotherapy responses. In addition, the combination of crotonate with chemotherapy or radiotherapy will be worthy of future investigation in breast cancer treatment.

Gut microbiome exerts a profound impact on breast cancer progression (*41*). In addition, modulation of gut microbiota and their metabolites influences therapeutic response. We observed that antibiotic-induced disruption of gut microbiota reduces crotonyl-CoA production, thereby promoting tumor growth and metastasis. However, when exogenous crotonate was administered to germ-free mice, both tumor growth and metastasis were markedly suppressed (Fig. 8, A to E), highlighting a gut microbiota–crotonate–crotonyl-CoA axis regulating breast cancer progression. Specific subsets of anaerobic bacteria, particularly members of the *Clostridium*, *Eubacterium*, and *Butyrivibrio* genera, produce SCFAs in the intestine (*42*). For example, clostridial clusters IV and XIVa are butyrate producers. However, the specific bacterial flora responsible for producing crotonate remain unknown. Future investigations can focus on the gut microbiota that produces crotonate.

In summary, we demonstrate the vital role of crotonate in blocking breast cancer metastasis. Combined administration of crotonate and anti-PD-L1 antibody promotes breast cancer responses to immunotherapy. Thus, crotonate is a valued drug candidate for breast cancer therapy.

## MATERIALS AND METHODS

### Cell culture

Human breast cancer cell line MDA-MB-231 was cultured in Leibovitz’s L-15 medium (catalog no. BL313A, Biosharp), supplemented with 10% fetal bovine serum (FBS) (Invitrogen), at 37°C under 0.5% CO_2_ in a humidified incubator. Human normal breast epithelial cell line MCF10A was maintained in Dulbecco’s modified Eagle’s medium (DMEM)/F12 medium (catalog no. CM10092, Macgene) supplemented with 10% FBS. Mouse breast cancer cell line Py8119 was cultured in Ham’s F12K medium (catalog no. GUMD-B307, HyCyte) containing 5% FBS. Mouse breast cancer cell line 4T1 and human breast cancer cell line T47D were grown in RPMI 1640 medium (catalog no. CM10041, Macgene) supplemented with 10% FBS. Other cell lines [SUM159, MCF7, Hs 578T, and human embryonic kidney (HEK) 293T] were maintained in high-glucose DMEM (catalog no. CM10013, Macgene) supplemented with 10% FBS. All cells were incubated at 37°C under 5% CO_2_ in a humidified incubator. Penicillin (100 units/ml) and streptomycin (0.1 mg/ml) were added to all culture media to prevent microbial contamination.

### Nontargeted metabolomics

MCF10A and MDA-MB-231 cells were collected for nontargeted metabolomics analysis. The samples were subjected to ultrasonic extraction in an ice-water bath. Following centrifugation at 4°C (13,000 rpm), the supernatant was transferred to a glass sampling vial, dried using a freeze concentration centrifugal dryer, and further vacuum-dried at room temperature. Methoxylamine hydrochloride in pyridine, N,O-Bis (trimethylsilyl)trifluoroacetamide (BSTFA), and *n*-hexane were used to derivatize the extract prior to gas chromatography–mass spectrometry analysis. The derivatized samples were analyzed using an Agilent 7890B gas chromatography system coupled with an Agilent 5977A MSD system (Agilent Technologies Inc., CA, USA). Metabolic differences between sample groups were subsequently identified and analyzed.

### Lipid droplet staining

Two thousand cells were seeded into a glass-bottomed cell culture microplate. The cells were gently washed twice with PBS, followed by the addition of BODIPY 493/503 working solution (10 μM; catalog no. D3922, Invitrogen) together with Hoechst 33342 (catalog no. R37165, Invitrogen). The cells were then incubated at room temperature for 10 min. After two gentle washes with PBS, fluorescence images were captured using a confocal microscope (Zeiss LSM 780 with Airyscan or Nikon) and analyzed with Zen Blue software.

### WST-1 and colony formation

Cell viability was assessed using the WST-1 assay. Cells were seeded into 96-well plates at a density of 2000 cells per well. The WST-1 working solution was added according to the manufacturer’s instructions, and the plates were then returned to the humidified incubator for ~2 hours. The absorbance at 450 nm (OD_450_) was subsequently measured using a microplate reader (Tecan, Switzerland). For the colony formation assay, 500 cells per well were seeded into six-well plates. After 2 weeks, the cells were fixed with 4% paraformaldehyde, stained with 0.1% crystal violet, and imaged.

### Transwell assay

MDA-MB-231 cells (5 × 10^4^) or SUM159/MCF7 cells (1 × 10^5^) were seeded into a Transwell chamber (catalog no. 353097, BD, USA) with 8-μm pores, containing 0.1 ml of culture medium supplemented with 0.1% FBS. The chamber was then placed into a 24-well plate containing 0.8 ml of culture medium supplemented with 20% FBS. Following incubation for 15 hours (MDA-MB-231), 9 hours (SUM159), or 30 hours (MCF7), the chamber was fixed with 4% paraformaldehyde and stained with 0.1% crystal violet, and the inner side of the membrane was gently wiped with a cotton swab to remove nonmigrated cells. Five random fields from the bottom side of each group were captured under a microscope, and the number of migrated cells was quantified using ImageJ software.

### TAXIScan-FL assay

The cells were digested when they were in the exponential growth phase, and the cell concentration was adjusted to 1 × 10^6^ cells/ml. The TAXIScan-FL cell dynamic analysis system was used to record the cell movement trajectories. A concentration gradient was formed using 40% FBS as the chemoattractant to stimulate cell movement. The recording lasted for 5 to 6 hours, and then, the parameters, such as cell migration distance and movement speed, were analyzed.

### Cell transfection

Plasmids were transfected using polyethylenimine or Lipofectamine 2000/3000 transfection reagent. Cells were collected 48 to 96 hours after transfection for subsequent experiments. All small interfering RNAs (siRNAs) were purchased from Guangzhou RiboBio Co., Ltd. and transfected using RNAiMAX. Cell samples were collected 48 to 72 hours after transfection for subsequent analysis.

### RNA sequencing

Basic quality control was performed on the resulting FASTQ files using FastQC. Raw FASTQ files were trimmed with fastp and aligned to the hg38 human reference genome using HISAT2 under default parameters. Gene expression levels were quantified from BAM files using FeatureCounts. Differentially expressed genes were identified using EdgeR. Functional enrichment analysis, including Gene Ontology (GO) and KEGG pathway enrichment, was performed and visualized with ClusterProfiler. GSEA was performed using enrichplot.

### CUT&Tag

Basic quality control was performed on the resulting FASTQ files using FastQC. Raw FASTQ files were trimmed with fastp and aligned to the hg38 human reference genome using Bowtie2 under default parameters. SAMtools was used to sort and index the BAM files. Peak calling was conducted using MACS2 with either the wide peak mode. To facilitate subsequent visualization of the chromatin immunoprecipitation sequencing (ChIP-seq) signal, we generated bigWig files using the Deeptools bamCoverage script. Heatmaps and profile plots were created with Deeptools. Annotation of ChIP-seq peaks was carried out using the R package ChIPseeker. Functional enrichment analysis of target genes, including GO and KEGG pathway enrichment, was performed and visualized using ClusterProfiler.

### RNA extraction and quantitative real-time polymerase chain reaction

Total RNA was extracted from cells using TRIzol reagent (Invitrogen) according to the manufacturer’s instructions. cDNA was synthesized using a HiScript II Q RT SuperMix Kit (Vazyme) according to the manufacturer’s instructions. Quantitative real-time polymerase chain reaction (qPCR) was performed using a ChamQ SYBR qPCR Master Mix (Vazyme) by LightCycler 96 Instrument (Roche).

### Protein extraction, WB, and coimmunoprecipitation

Cells or tissues were lysed for 30 min using a RIPA buffer (1 × PBS, pH 7.4, 0.5% sodium deoxycholate, 1% NP40, and 0.1% SDS) containing the cocktail inhibitor (Roche Basel) and centrifuged at 4°C for 15 min (cells) or 30 min (tissues). The supernatants were taken, and the protein was quantified using the bicinchoninic acid kit (Thermo Fisher Scientific). Loading buffer (5×) was added to the lysates and heated at 100°C for 5 to 10 min, and SDS–polyacrylamide gel electrophoresis (SDS-PAGE) was then performed. Polyvinylidene difluoride films were immunoblotted with primary antibodies and horseradish peroxidase (HRP)–conjugated secondary antibodies, and the proteins were then detected using enhanced chemiluminescence. For coimmunoprecipitation (Co-IP) assay, control immunoglobulin G and 25 to 40 μl of protein A agarose were used to preclear the protein lysis at 4°C for 2 hours. Then, the solution was centrifuged, and the supernatants were taken to be incubated overnight at 4°C with antibodies. Protein A agarose (25 to 40 μl) was added to the lysates and incubated at 4°C for 4 to 6 hours. The beads were washed using precooled RIPA buffer for five times and then detected by WB assay.

### Mass spectrometry

FLAG-EZH2 was transfected into MCF7 cells and treated with crotonate (20 mM) for 48 hours. Whole-cell protein lysates were extracted and enriched by M2 agarose gel. After SDS-PAGE gel electrophoresis, the protein was stained with Coomassie blue. Bands ranging from 70 to 130 kDA were cut for mass spectrometry. Crotonylation sites of EZH2 were analyzed.

### H3K27 HMT activity

The EpiQuik Histone Methyltransferase Activity/Inhibition Assay Kit (H3K27) (catalog no. P-3005, EPIGENTEK) was used for measuring HMT’s activity that target H3K27. In this kit, the histone substrate was stably captured on the strip wells through biotin-streptavidin binding. The nuclear extracts were added to each well, and the strip wells were incubated at 37°C for 60 min. During the incubation, HMT would transfer methyl groups to histone H3 to methylate the substrate at K27. An HRP-conjugated antibody-color development system was used to quantify the ratio or amount of methylated H3K27, which was directly proportional to enzyme activity. The HMT activity was calculated based on the amount of methylated H3K27 converted by the HMTs: HMT activity (OD/hour per milligram) = OD (sample − blank)/[protein amount (milligram) × incubation time (hour)].

### Multiple immunofluorescence staining

Human breast cancer tissue microarrays were purchased from the National Human Genetic Resources Sharing Service Platform 2005DKA21300 (Shanghai Outdo Biotechnology Company Ltd., China) (permit number: SHYJS-CP-1807002). Multiple immunofluorescence staining was performed according to the instructions of the TissueGnostics Multiple IHC Assay Kit (TissueGnostics, TGFP550). Images were captured using the Axioscan 7 (Zeiss) and were analyzed by Zen Blue software.

### Animal model

C57BL/6N, BALB/c, and BALB/c nude mice (4 to 6 weeks old) were purchased from the Animal Experimental Center of Peking University Health Science Center and raised in a specific pathogen-free animal laboratory. The Ethics Committee of Peking University Health Science Center approved the mouse experiments (permit number: BCJB0071) for this study. The mice were handled in accordance with the ethical standards of the Helsinki Declaration of 1975 and the revised version in 1983. The models we used in this study are presented below.

#### Xenografts in mice

MDA-MB-231 cells were orthotopically inoculated onto the right fourth abdominal mammary fat pad of BALB/c nude mice (5 × 10^6^ cells each mouse), and 4T1 cells onto BALB/c mice and Py8119 cells onto C57BL/6N mice (5 × 10^5^ cells each mouse). About several days later, when most of the mice could be observed with visible tumors, the tumor-forming mice were divided evenly into different groups based on the size of the tumors. The volume of each tumor (*V* = length × width × height × 0.5mm^3^) and the mice weight were measured on a regular basis. After the mice were sacrificed, all the tumors were dissociated from the mice and photographed.

#### Teil vain injection model

MDA-MB-231-luci cells were inoculated into the teil vain of each BALB/c nude mice (8 × 10^5^ cells each mouse). Py8119-luci cells were inoculated into the same place of C57BL/6N mice (5 × 10^5^ cells each mouse). After 2 (C57BL/6N) or 6 (BALB/c nude) weeks, d-luciferin potassium salt was injected into the peritoneal cavity of each mouse, and the luciferase signal was detected by IVIS Spectrum (PerkinElmer).

#### Left cardiac ventricle injection model

MDA-MB-231-luci cells were inoculated into the left cardiac ventricle of each BALB/c nude mice (1 × 10^4^ cells each mouse) through VisualSonics Vevo3100 (Fujifilm). Py8119-luci cells were inoculated into the same place of C57BL/6N mice (1 × 10^4^ cells each mouse). After 2 to 4 weeks, d-luciferin potassium salt was injected into the peritoneal cavity of each mouse, and the luciferase signal was detected by IVIS Spectrum (PerkinElmer).

### Abx treatment

Mice with intestinal microbiota deficiency were constructed through quadruple antibiotics (Abx) treatment. Vancomycin (1 mg/ml), ampicillin (2 mg/ml), neomycin (2 mg/ml), and metronidazole (2 mg/ml) were administered by intragastric administration for the first time, and then, the mice were fed with water containing vancomycin (0.5 mg/ml), ampicillin (1 mg/ml), neomycin (1 mg/ml), and metronidazole (1 mg/ml) for 2 weeks. Then, the mice were grouped for the subsequent experiments.

### Crotonyl-CoA detection

Crotonyl-CoA was detected by the Metabolomics Platform at Peking University Health Science Center. The tumor tissues from mice were homogenized and mixed with methanol/water (v/v = 1/1) at a ratio of 1:40. After ultrasonic treatment, the mixture was centrifuged at low temperature. The supernatant was collected and concentrated at low temperature (<10°C). Subsequently, 30 μl of methanol/acetonitrile (v/v = 1/1) was added followed by ultrasonic treatment for 1 min, and then, 20 μl of H_2_O was added. Ultrasonic treatment was then performed for another 1 min. After low-temperature centrifugation, the supernatant was collected. The metabolites were analyzed by ultrahigh-performance liquid chromatography, and the chromatographic peak of crotonyl-CoA was integrated. Quantitative analysis was performed using the external standard method.

### Metagenomic sequencing

The feces of C57BL/6N mice were collected, and microbial genomic DNA was extracted from the samples. A DNA library was constructed and subjected to metagenomic sequencing analysis using Illumina PE150. Differential gene expression was evaluated between groups. Subsequently, species annotation was performed to identify microbial taxa, and intergroup differences in microbial species composition were analyzed. Functional enrichment analysis was conducted on both the differentially expressed genes and the identified microbial species.

### Reagents

Crotonate (20 to 60 mg/kg, once every 2 days), crotonyl-CoA (60 μg/kg, once every 2 days), tazemetostat (200 mg/kg, once every 4 days), and tucidinostat (25 mg/kg, once every 2 days) were administered by intragastric administration, InVivoMAb anti-mouse PD-L1 (300 μg per mouse, once every 4 days) was administered by intraperitoneal injection.

### Flow cytometry

Different breast cancer cell lines or tumors from BALB/c mice were dissociated into single cells. After filtered by 70-mm filters, cells were incubated with antibodies for 30 min at 4°C. We used a flow cytometer to analyze the cell samples and FlowJo software to analyze experimental results.

### Statistical analysis

We used GraphPad Prism software to perform all the statistical analyses. All experimental results were presented as means ± SD or means ± SEM. We used Mann-Whitney test or Welch’s correction test to compare two groups and two-way analysis of variance (ANOVA) to analyze multiple groups. Statistical significance was considered when *P* < 0.05 (*).

## Supplementary Materials

### This PDF file includes:

Figs. S1 to S9 Table S1 Uncropped Western blots
