# Supplementary material for: Crotonate suppresses breast cancer metastasis and promotes immunotherapy response by inducing ACSS2-mediated EZH2-K348 crotonylation
Source: Sci Adv. 2026 Jan 16;12(3):eaea9892. doi: 10.1126/sciadv.aea9892 (PMC12810637; doi:10.1126/sciadv.aea9892)
Supplement: Supplementary file 1 — Figs. S1 to S9 Table S1 Uncropped Western blots [file sciadv.aea9892_sm.pdf]

Science Advances
AAAS

Supplementary Materials for

Crotonate suppresses breast cancer metastasis and promotes immunotherapy response by inducing ACSS2-mediated EZH2-K348 crotonylation

Bo Liu *et al.*

Corresponding author: Yu Yu, yuyu@bjmu.edu.cn; Hongquan Zhang, hongquan.zhang@bjmu.edu.cn

Sci. Adv. **12**, eaea9892 (2026)
DOI: 10.1126/sciadv.aea9892

This PDF file includes:

Figs. S1 to S9
Table S1
Uncropped Western blots

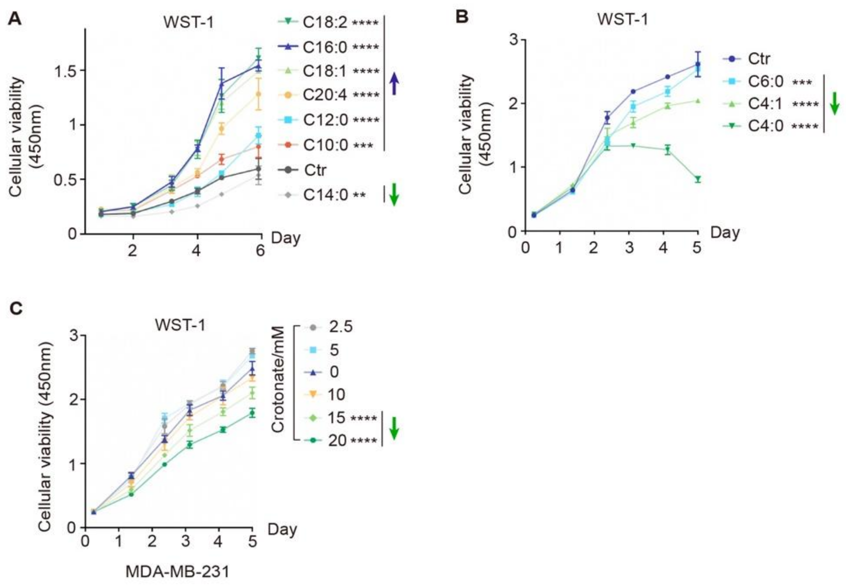

Fig. S1. Crotonate suppresses breast cancer proliferation. (A-B) MDA-MB-231 cells were treated with fatty acids for 72hr, and followed by WST-1. Data represent the mean ± SD. **p < 0.01, ***p < 0.001, ****p < 0.0001 by Two-way ANOVA. (C) MDA-MB-231 cells were treated with different concentrations of crotonate for 72hr, followed by WST1. Data represent the mean ± SD. ****p < 0.0001 by Two-way ANOVA.

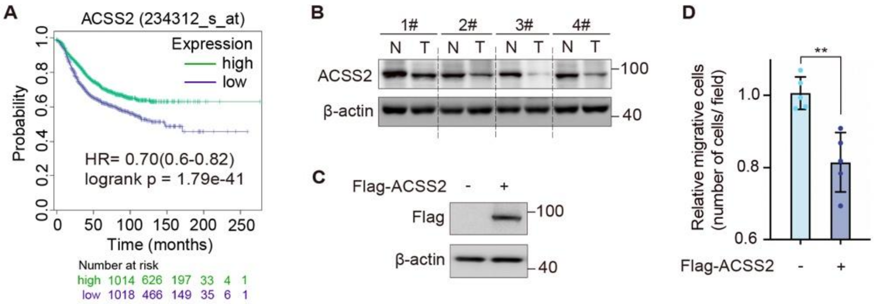

Fig. S2. The level of ACSS2 in tumors. (A) Kaplan-Meier survival analysis of ACSS2 expression and overall survival in breast cancer patients. ****p < 0.0001 by Log-Rank test. (B) Total protein from tumor tissues and adjacent normal tissues were extracted from MMTV-PyMT mice, followed by WB. (C) WB analysis of the efficiency of ACSS2 overexpression. (D) SUM159 cells were overexpressed Flag-ACSS2 and followed by transwell assay. Data represent the mean ± SD. **p < 0.01 by unpaired t test with Welch's correction.

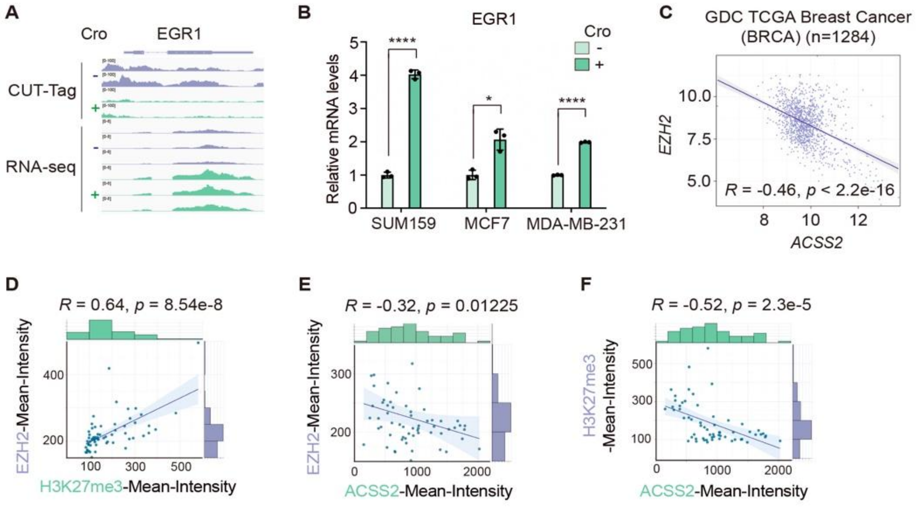

Fig. S3. H3K27me3 occupancy on representative genes.

(A) CUT&Tag and RNA-seq genomic tracks of EGR1. (B) qPCR detecting the mRNA level of EGR1 in breast cancer cells treated by crotonate. (C) Correlation analysis was conducted on the mRNA levels of ACSS2 and EZH2 in breast cancer patients from the TCGA database. (D-F) Correlation analysis of EZH2, H3K27me3, and ACSS2 levels in Fig. 5J.

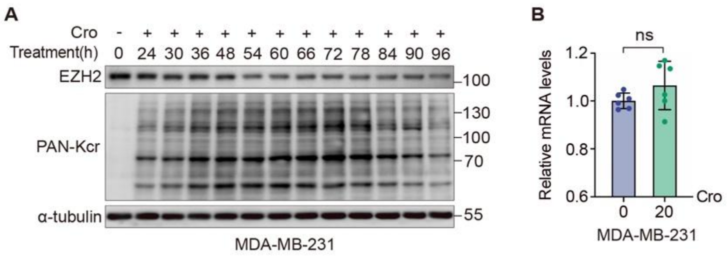

Fig. S4. Crotonate regulates EZH2 level. (A) MDA-MB-231 cells were treated with crotonate (20mM) for different time, followed by WB. (B) The MDA-MB-231 cells were treated with 20mM crotonate for 72hr, and then the mRNA expression level of EZH2 was detected by q-PCR.

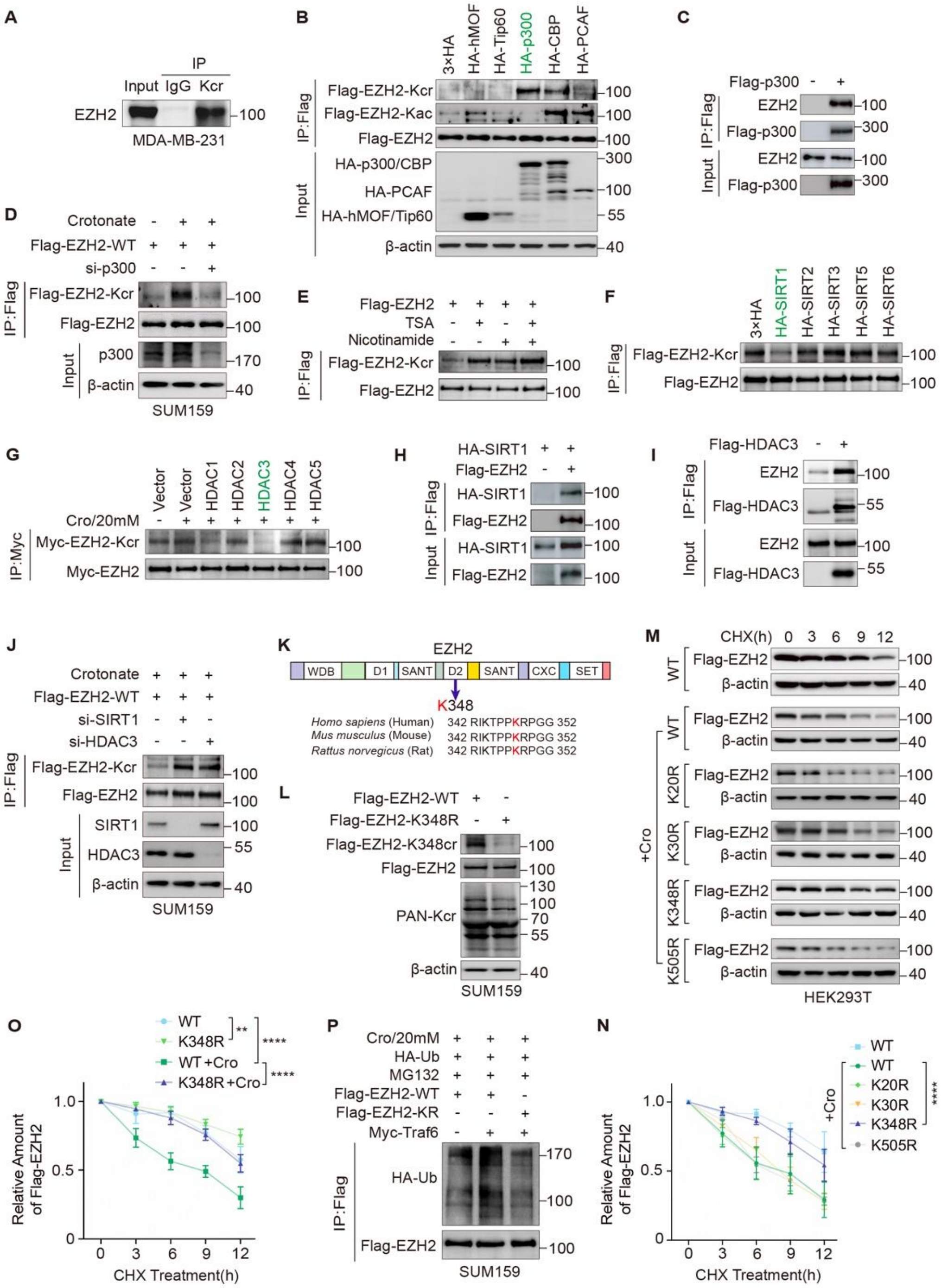

A
IP
Input
IgG
Kcr
EZH2
100
MDA-MB-231
B
3×HA
HA-hMOF
HA-Tip60
HA-p300
HA-CBP
HA-PCAF
IP:Flag
Flag-EZH2-Kcr
Flag-EZH2-Kac
Flag-EZH2
Input
HA-p300/CBP
HA-PCAF
HA-hMOF/Tip60
β-actin
300
100
55
40
C
Flag-p300
IP:Flag
EZH2
Flag-p300
Input
D
Crotonate
Flag-EZH2-WT
si-p300
Flag-EZH2-Kcr
Flag-EZH2
p300
β-actin
170
SUM159
E
TSA
Nicotinamide
F
HA-SIRT1
HA-SIRT2
HA-SIRT3
HA-SIRT5
HA-SIRT6
G
Vector
HDAC1
HDAC2
HDAC3
HDAC4
HDAC5
Cro/20mM
IP:Myc
Myc-EZH2-Kcr
Myc-EZH2
H
HA-SIRT1
Flag-EZH2
I
Flag-HDAC3
J
si-SIRT1
si-HDAC3
SIRT1
HDAC3
K
EZH2
WDB
D1
SANT
D2
CXC
SET
K348
Homo sapiens (Human) 342 RIKTPPKRPGG 352
Mus musculus (Mouse) 342 RIKTPPKRPGG 352
Rattus norvegicus (Rat) 342 RIKTPPKRPGG 352
L
Flag-EZH2-K348R
Flag-EZH2-K348cr
PAN-Kcr
130
70
M
CHX(h)
0
3
6
9
12
WT
K20R
K30R
K348R
K505R
+Cro
HEK293T
O
WT
K348R
WT+Cro
K348R+Cro
**
****
Relative Amount of Flag-EZH2
CHX Treatment(h)
1.0
0.5
0
P
HA-Ub
MG132
Flag-EZH2-KR
Myc-Traf6
N

Fig. S5. EZH2 crotonylation at K348 regulates protein stability. (A) MDA-MB-231 cells were treated with TSA (3μM) and Nicotinamide (5mM) for 12hr, followed by Co-IP. (B) Flag-EZH2 and five acyltransferases HA-hMOF, HA-Tip60, HA-p300, HA-CBP and HA-PCAF were co-transfected into HEK293T cells, followed by Co-IP. (C) Flag-p300 was transfected into HEK293T cells, followed by Co-IP to test the interaction between p300 and EZH2. (D) WT analysis of Flag-EZH2-Kcr levels in SUM159 cells with p300 knockdown. (E) Flag-EZH2 was transfected into HEK293T cells. 48hr later, cells were treated with TSA (3μM) and/or Nicotinamide (5mM) for 12hr, followed by Co-IP. (F-G) Flag-EZH2 and different deacyltransferases (SIRTs and HDACs) were co-transfected into HEK293T cells, together with crotonate treatment (20mM, 48hr), followed by Co-IP. (H) Flag-EZH2 and HA-SIRT1 were co-transfected into in HEK293T cells, followed by Co-IP to test their interaction between EZH2 and SIRT1. (I) Flag-HDAC3 was transfected into HEK293T cells, followed by Co-IP to test the interaction between EZH2 and HDAC3. (J) WB analysis of Flag-EZH2-Kcr level in SUM159 cells with SIRT1and HDAC3 knockdown. (K) The K348 residue in EZH2 is evolutionarily conserved in the indicated species. (L) WB analysis of EZH2-K348cr and pan-Kcr levels in SUM159-EZH2-WT and K348R stable cells. (M-N) Flag-EZH2-WT or four mutants were transfected into HEK293T cells. After 12hr, cells were treated with crotonate (20mM, 48hr), together with CHX (100μg/mL), followed by WB. The protein levels of Flag-EZH2 were normalized to β-actin level at different time point from three independent experiments. (O) The protein levels of Flag-EZH2 in Fig. 5I were normalized to β-actin level at different time points from three independent experiments. (P) The indicated plasmids were transfected into SUM159 cells to detect the ubiquitination of Flag-EZH2.

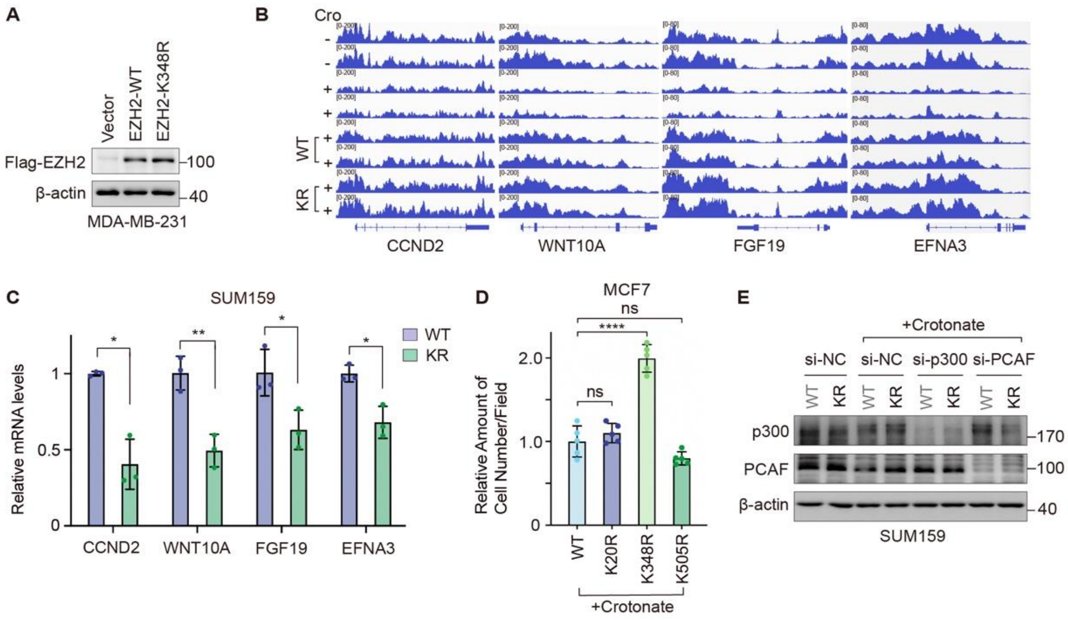

Fig. S6. Representative genes regulated by EZH2-K348 crotonylation. (A) WB analysis of Flag-EZH2 level in stable cells. (B) CUT&Tag genomic tracks of representative genes. (C) qPCR detecting the mRNA level of representative genes in EZH2-WT and K348R stable cells. (D) FlagEZH2-WT, K20R, K348R, and K505R were overexpressed in MCF7 cells and then treated the cells with crotonate (20mM) for 72hr, followed by transwell assay. Data represent the mean ± SD. ****$p < 0.0001$ by unpaired t test with Welch's correction. (E) WB analysis of the efficiency of p300 and PCAF kncokdown.

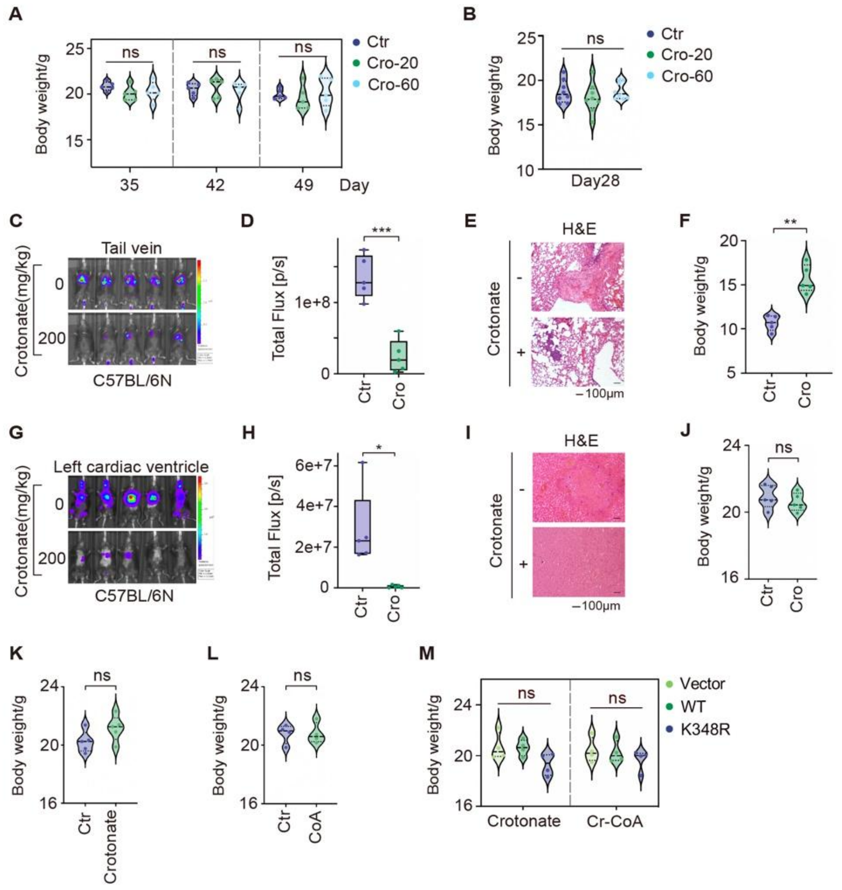

Fig. S7. Crotonate inhibits breast cancer cell metastasis in vivo. (A-B) The mice weights in Fig.6A-H. (C-F) Py8119-luci cells were injected into the tail vein of C57BL/6N mice. Then the mice were divided into 2 groups (n=5/group) and given saline or crotonate (200mg/kg) by gavage every two days. IVIS imaging on 14th day (C), the fluorescence intensity (D), HE staining of the lung tissues (E), mice weights (F). $^{**}p < 0.01$, $^{***}p < 0.001$ by unpaired t test with Welch's correction. (G-J) Py8119-luci cells were injected into the left cardiac ventricle of C57BL/6N mice. Mice were divided into 2 groups (n=5/group) and given saline or crotonate (200mg/kg) by gavage every two days. IVIS imaging on 14th day (G), the fluorescence intensity (H), HE staining of the liver tissues (I) and mice weights (J). $^{*}p < 0.05$ by unpaired t test with Welch's correction. (K-M) The mice weights in Fig. 6I, Fig. 6L, and Fig. 6R.

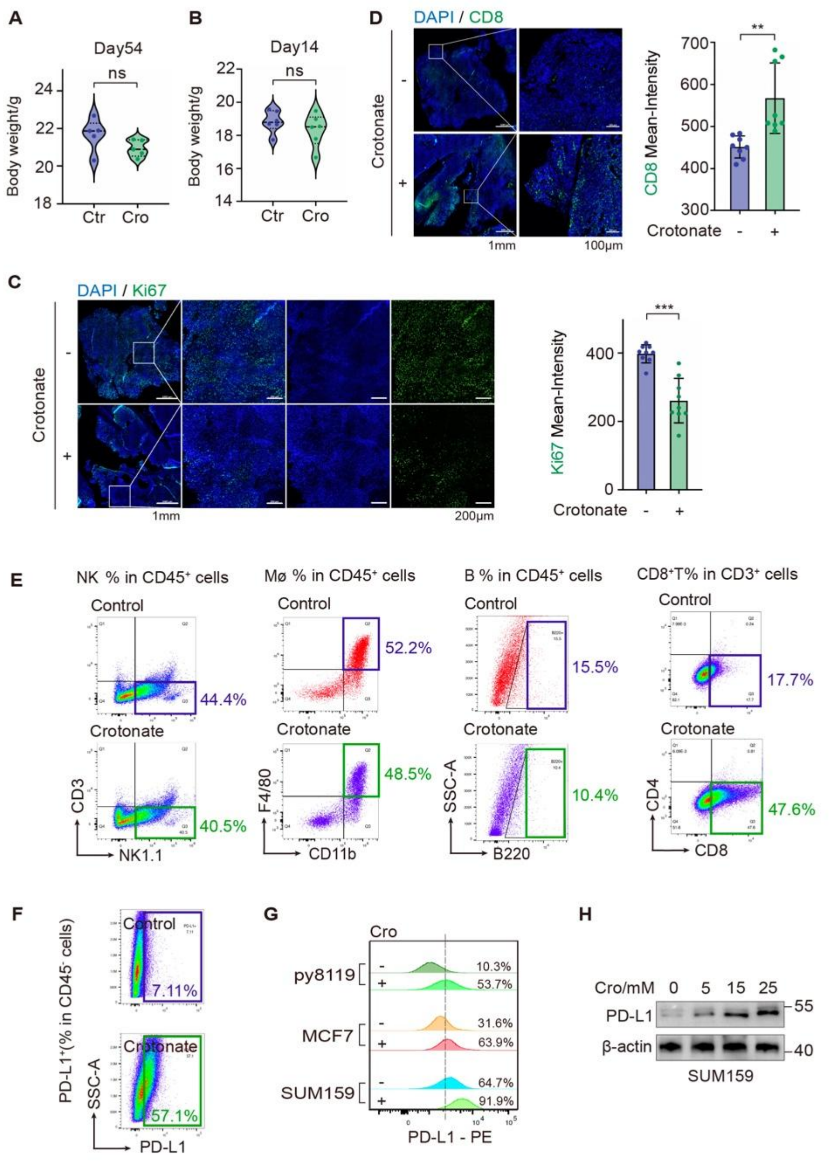

Fig. S8. Combination of crotonate and anti-PD-L1 Mab. (A-B) The mice weights in Fig.7A and Fig. 7D. (C-F) Tumor tissues in Fig. 7D were performed immunofluorescence staining using the nuclei (DAPI, blue), Ki67 (Alexa Flour 488, green) (C) and CD8 (Alexa Flour 488, green) (D), and flow cytometry analysis (E-F). Data represent the mean ± SD. $**p < 0.01$, $***p < 0.001$ by unpaired t test with Welch's correction. (G) Flow cytometry analysis of breast cancer cells treated by crotonate. (H) SUM159 cells were treated with crotonate (0,5,15,25mM) for 72hr, followed by WB.

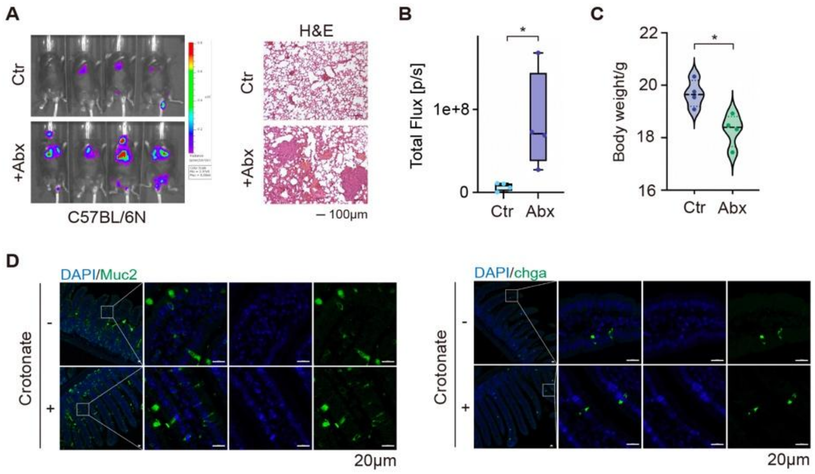

Fig. S9. Abx treatment in C57BL/6N mice. (A-C) The C57BL/6N mice were divided into 2 groups and administrated a four-drug antibiotic (Abx) treatment. After 14 days, py8119-luci cells were injected into the tail vein of the mice. Another 14 days later, IVIS imaging, HE staining of the lung tissues (A), the fluorescence intensity (B) and the mice weights (C) were shown. *$p < 0.05$ by unpaired t test with Welch's correction. (D) Immunofluorescence staining was performed using the nuclei (DAPI, blue), Muc2 (Alexa Flour 488, green) and chga (Alexa Flour 488, green).

REAGENT or RESOURCE	SOURCE	IDENTIFIER
**Antibodies**		
Rabbit monoclonal anti-H3K27me3 (C36B11)	Cell Signaling Technology	Cat# 9733, RRID:AB_2616029
Rabbit monoclonal anti-EZH2 (D2C9) XP®	Cell Signaling Technology	Cat# 5246, RRID:AB_10694683
Rabbit monoclonal anti-H3 (D1H2) XP®	Cell Signaling Technology	Cat# 4499, RRID:AB_10544537
Rabbit monoclonal anti-PCAF(C14G9)	Cell Signaling Technology	Cat# 3378, RRID:AB_2128409
Rabbit monoclonal anti-p21 Waf1/Cip1 (12D1)	Cell Signaling Technology	Cat# 2947, RRID:AB_823586
Rabbit polyclonal anti-H3K27me3	Merck Millipore	Cat# 07-449, RRID:AB_310624
Mouse monoclonal anti-Actin (2Q1055)	Santa Cruz Biotechnology	Cat#sc-58673,RRID:AB_2223345
Rabbit polyclonal anti-PD-L1 (C-terminal)	Proteintech	Cat# 28076-1-AP, RRID:AB_2881052
Mouse monoclonal anti-Myc-Tag (9E10)	Abcam	Cat# ab32, RRID:AB_303599
Rabbit polyclonal anti-HA tag	Abcam	Cat# ab9110, RRID:AB_307019
Rabbit monoclonal anti-KAT3B/p300	Abcam	Cat# ab259330, RRID:AB_3676060
Mouse monoclonal anti-SIRT1 (19A7AB4)	Abcam	Cat# ab110304, RRID:AB_10864359
Rabbit monoclonal anti-HDAC3 (E19J18)	Selleck	Cat# F0838
Rabbit polyclonal anti-Crotonyllysine	PTM-BIO	Cat# PTM-501, RRID:AB_2877694
Rabbit polyclonal anti-Crotonyl-EZH2-K348	PTM-BIO	Cat# CP0405
Mouse monoclonal anti-His-Tag	EASYBIO	Cat# BE2062
Mouse monoclonal anti-GAPDH	ZSGB-BIO	Cat# TA-08, RRID:AB_2747414
Mouse monoclonal anti-FLAG M2	Sigma-Aldrich	Cat# F1804, RRID:AB_262044
Rabbit polyclonal anti-ACSA (ACSS2)	Affinity	Cat# DF3728, RRID:AB_2836092
*InVivo*MAb anti-mouse PD-L1 (B7-H1)	BioXCell	Cat# BE0101, RRID:AB_10949073
7-AAD Viability Staining Solution	Biolegend	Cat# 420404
BV605 anti-mouse CD45	Biolegend	Cat# 103140, RRID:AB_2562342
FITC anti-mouse CD3	Biolegend	Cat# 100203, RRID:AB_312660
APC/Cyanine7 anti-mouse CD4	Biolegend	Cat# 100413, RRID:AB_312698
PE/Cyanine7 anti-mouse CD8a	Biolegend	Cat# 100722, RRID:AB_312761
PE anti-mouse NK1.1	Biolegend	Cat# 156504, RRID:AB_2783136
FITC anti-mouse F4/80	Biolegend	Cat# 123107, RRID:AB_893500
PE anti-mouse/human CD11b	Biolegend	Cat# 101207, RRID:AB_312790
PE anti-mouse CD274 (PD-L1)	Biolegend	Cat# 124308, RRID:AB_2073556
PE anti-human CD274 (PD-L1)	Biolegend	Cat# 329706, RRID:AB_940368
**Bacterial and Virus Strains**		

DH5 Escherichia coli	Tiangen	CB101
**Chemicals,Peptides,and Recombinant Proteins**		
Lipofectamine® RNAiMAX Transfection Reagent	Thermo Fisher Scientific	Cat# 13778150
Lipofectamine® 2000 Transfection Reagent	Thermo Fisher Scientific	Cat# 11668019
Lipofectamine® 3000 Transfection Reagent	Thermo Fisher Scientific	Cat# L3000001
DAPI	Macgene	Cat# CD051
Dynabeads™ Protein A	Invitrogen	Cat# 10002D
Trizol	Invitrogen	Cat# 15596018
cOmplete ULTRA Tablets, Mini, EASYpack Protease	Roche	Cat# 000 000005892970001
Cell Proliferation Reagent WST-1	Roche	Cat# 11644807001
DEPC	Amresco	Cat# E174
2×ChamQ SYBR qPCR Master Mix	Vazyme	Cat# Q311-02
HiScript II Q RT SuperMix	Vazyme	Cat# R233-01
Puromycin dihydrochloride	Applichem	Cat# A2856.0010
G418	Gibco	Cat# 11811-031
UltraPure™ Agarose	Invitrogen	Cat# 16500100
HEPES	Sigma-Aldrich	Cat# H3375
Cycloheximide (CHX)	Sigma-Aldrich	Cat# C7698
2-Butenoyl (Crotonoyl) coenzyme A lithium salt	Sigma-Aldrich	Cat# C6146
Crotonic acid	Sigma-Aldrich	Cat# 107-93-7
Tazemetostat	MCE	Cat# HY-13803
Tucidinostat	MCE	Cat# HY-109015
MG132	Selleck	Cat# S2619
D-Luciferin, Potassium Salt	Yeasen	Cat# 40902ES03
Polyethyleneimine (PEI)	Polyscience	Cat# 24765
TWEEN20	Amresco	Cat# 0777
Hoechst 33342 Ready Flow	Invitrogen	Cat# R37165
BODIPY 493/503	Invitrogen	Cat# D3922
**Critical Commercial Assays**		
NovoNGS® CUT&Tag 4.0 High-Sensitivity Kit (for	Novoprotein	Cat# N259-YH01
Mut Express II Fast Mutagenesis Kit V2	Vazyme	Cat# C214-01
EpiQuik Histone Methyltransferase Activity/Inhibition	EPIGENTEK	Cat# P-3005
**Experimental Models:Strains/Organisms**		

Mouse: Female C57BL/6N	Laboratory Animal Center of Peking University Health Science Center	N/A
Mouse: Female BALB/c-nu	Laboratory Animal Center of Peking University Health Science Center	N/A
Mouse: Female BALB/c	Laboratory Animal Center of Peking University Health Science Center	N/A
**Recombinant DNA**		
p3×FLAG-CMV10	This paper	N/A
p3×FLAG-CMV10-EZH2	This paper	N/A
p3×FLAG-CMV10-EZH2-K20R	This paper	N/A
p3×FLAG-CMV10-EZH2-K30R	This paper	N/A
p3×FLAG-CMV10-EZH2-K348R	This paper	N/A
p3×FLAG-CMV10-EZH2-K505R	This paper	N/A
p3×FLAG-CMV10-HDAC1,2,3,4,5	This paper	N/A
pCMV6-AC-HA	This paper	N/A
pCMV6-AC-HA-CBP	This paper	N/A
pCMV6-AC-HA-p300	This paper	N/A
pCMV6-AC-HA-PCAF	This paper	N/A
pCMV6-AC-HA-hMOF	This paper	N/A
pCMV6-AC-HA-Tip60	This paper	N/A
pCMV6-AC-HA-Sirt1,2,3,5,6	This paper	N/A
HA-Ub	This paper	N/A
pLVX-AcFLAG1-N1	This paper	N/A
pLVX-IRES-Puro-3×FLAG-ACSS2	This paper	N/A
pMD2.G	Addgene	Cat# 12259
psPAX2	Addgene	Cat# 12260
**Software and Algorithms**		
GraphPad Prism 9.0	GraphPad Software	http://www.graphpad.com/scientific-software/prism/
**qRT-PCR**		

Gene	Forward	Reverse
Human *β-actin*	5’-GGCTGTGCTATCCCTGTACG-3’	5’-AGGTAGTCAGTCAGGTCCCG-3’

Human *EZH2*	5’-TGACTGCTTCCTACATCCTTTTC-3’	5’-TTGGTGGGGTCTTTATCCGC-3’
Human *ACSS2*	5’-GGATCACTGGTCATTCCTAC-3’	5’-GTGCTGTGTAGAACTTGGTC-3’
Human *CDKN1A*	5’-CGATGGAACTTCGACTTTGTCA-3’	5’-GCACAAGGGTACAAGACAGTG-3’
Human *CCND2*	5’-ACCTTCCGCAGTGCTCCTA-3’	5’-CCCAGCCAAGAAACGGTCC-3’
Human *EGR1*	5’-ACCCCTCTGTCTACTATTAAGGC-3’	5’-TGGGACTGGTAGCTGGTATTG-3’
Human *WNT10A*	5’-AGATCGCCATCCACGAATGC-3’	5’-ATCTTGTTGCGAGTCTCCAGG-3’
Human *FGF19*	5’-CGGAGGAAGACTGTGCTTTCG-3’	5’-CTCGGATCGGTACACATTGTAG-3’
Human *EFNA3*	5’-TCTCTGGGCTACGAGTTCCAC-3’	5’-CCTCAGACACTTCCAGTGCAG-3’
Human *GCDH*	5’-CGTCCCGAGTTTGACTGGC-3’	5’-GATGCGAGGCATGAGTCTCT-3’
Human *ECHS1*	5’-TCCTGACTGGAGCACCTTCT-3’	5’-GCATCTGTATGAAGGCAGCA-3’
Human *ACOX3*	5’-CTCGATGCCTACCGAGCAAG-3’	5’-TGGTTTTCTTAAAGCGGAGCA-3’
Human *ACADS*	5’-GGCGGCAGTTACACACCATC-3’	5’-GCGTAGGCCAGGTAATCGAG-3’
Human *ACOX1*	5’-CGCCGAGAGATCGAGAACATG-3’	5’-GCCAAACTCCCTCATCTTCTTCAC-3’
**siRNA**		
**Gene**	**siRNA sequence**	
Human *ACSS2*	CAGCAATGTTCTCCGAAAA	
	CCACATATCCGGACGTGAA	
	ACCCATGAAACCCGGTTCT	
Human *p300*	GGAUAAUGCCUAAUCAAGU	
	CCAUUAAGAGGAAGUUAGA	
	CAACCUAAGCACUGUUAGU	
Human *PCAF*	CCGUAUGUUCCCAUCUCAA	
	GCAAUUGGAUACUUUAAGA	
	GCUCGGUCUUCACUGUUAU	
Human *SIRT1*	GCAAAGGAGCAGAUUAGUA	
	GCAUCUUGCCUGAUUUGUA	
	GCGGGAAUCCAAAGGAUAA	
Human *HDAC3*	GCAUUGAUGACCAGAGUUA	
	GUGGUUAUACUGUCCGAAA	
	CUAGUGUCCAGAUUCAUGA	

Table S1.
KEY RESOURCES TABLE

Fig.4I

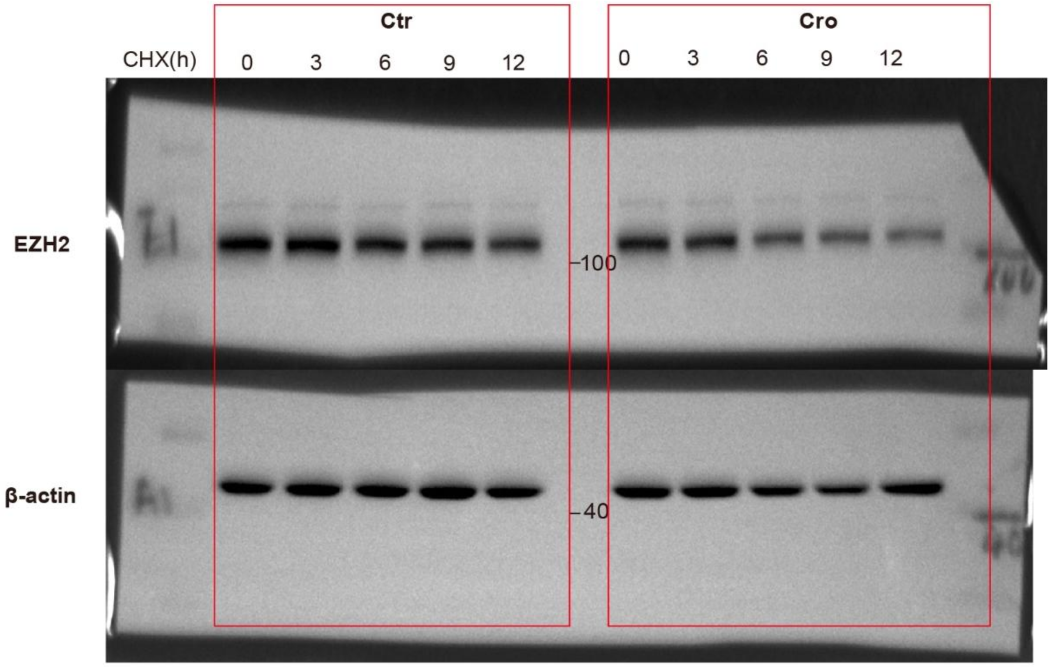

Fig. 4I Uncropped Western blot images
